# The effects of a prolonged exposure workshop with and without consultation on provider and patient outcomes: a randomized implementation trial

**DOI:** 10.1186/s13012-020-01014-x

**Published:** 2020-07-29

**Authors:** Edna B. Foa, Carmen P. McLean, Lily A. Brown, Yinyin Zang, David Rosenfield, Laurie J. Zandberg, Wayne Ealey, Brenda S. Hanson, Lora Rose Hunter, Ivett J. Lillard, Thomas J. Patterson, Julio Rosado, Valerie Scott, Charles Weber, Joseph E. Wise, Charles D. Zamora, Jim Mintz, Stacey Young-McCaughan, Alan L. Peterson, for the STRONG STAR Consortium

**Affiliations:** 1 Department of Psychiatry, School of Medicine, University of Pennsylvania, Philadelphia, PA 19104 USA; 2 National Center for PTSD, Dissemination and Training Division, VA Palo Alto Health Care System, Menlo Park, CA USA; 3 Department of Psychiatry and Behavioral Sciences, School of Medicine, Stanford University, Palo Alto, CA USA; 4 School of Psychological and Cognitive Sciences and Beijing Key Laboratory of Behavior and Mental Health, Peking University, Beijing, China; 5 Department of Psychology, Southern Methodist University, Dallas, TX USA; 6 Blanchfield Army Community Hospital, Fort Campbell, KY USA; 7 William Beaumont Army Medical Center, Fort Bliss, TX USA; 8 Landstuhl Regional Medical Center, Landstuhl, Germany; 9 Fielding Graduate University, California, Santa Barbara USA; 10 Department of Behavioral Health, Madigan Army Medical Center, Washington DC, USA; 11 South Texas Veterans Health Care System, San Antonio, Texas USA; 12 Washington Army National Guard, Washington, DC USA; 13 Evans Army Community Hospital, Fort Carson, CO USA; 14 Department of Psychiatry and Behavioral Sciences, School of Medicine, University of Texas Health Science Center at San Antonio, San Antonio, TX USA; 15 South Texas Veterans Health Care System, San Antonio, TX USA; 16 Department of Psychology, University of Texas at San Antonio, San Antonio, TX USA

**Keywords:** Prolonged exposure, PTSD, Provider training, Consultation

## Abstract

**Background:**

Prolonged exposure therapy (PE) is an evidence-based treatment for posttraumatic stress disorder (PTSD) that is underutilized in the military health system. Standard workshop training in PE may not be sufficient to alter provider behavior, but post-workshop consultation requires significant resources. Therefore, it is important to determine the incremental utility of post-workshop consultation.

**Methods:**

This study used a hybrid type III randomized implementation trial at 3 US Army installations. Providers were randomized to receive a 4-day prolonged exposure workshop (Standard training condition, *n* = 60), or the prolonged exposure workshop followed by 6–8 months of post-workshop expert case consultation (Extended training condition, *n* = 43). The effects training condition were examined on provider attitudes (self-efficacy in delivering PE, expectations for patient improvement, and beliefs about PE), use of PE and PE components, and clinical outcomes of patients with PTSD (using the Clinician-Administered PTSD Scale (CAPS-5)).

**Results:**

Extended condition providers reported greater improvements in self-efficacy, *b* = .83, 95% CI [.38, 1.27], *t*(79) = 3.71, *p* = .001, and *d* = .63. A greater proportion of patients in the Extended condition (44%) than in the Standard condition (27%) received at least 1 PE session, *b* = .76, *t*(233) = 2.53, *p* = .012, and OR = 2.13. Extended condition providers used more PE components (*M* = .9/session) than did Standard condition providers (*M* = .5/session), *b* = .54, 95% CI [.15, .93], *t*(68) = 2.70, *p* = .007, and *d* = .68. Finally, decrease in patients’ PTSD symptoms was faster for patients of Extended condition providers than for patients of Standard condition providers, *b* = − 1.81, 95% CI [− 3.57, − .04], *t*(263) = − 2.02, *p* = .045, and *d* = .66, and their symptoms were lower at the second assessment, *b* = − 5.47, 95% CI [− 9.30, − 1.63], *t*(210) = − 2.81, *p* = .005, and *d* = .66.

**Conclusions:**

Post-workshop consultation improved self-efficacy for delivering PE, greater use of PE, faster PTSD reduction, and lower PTSD severity at the second assessment. To our knowledge, this is the first demonstration that post-workshop case consultation for PE improves patient outcomes.

**Trial registration:**

Clinicaltrials.gov, NCT02982538. Registered December 5, 2016; retrospectively registered

Contributions to the literature

- Adding expert case consultation to workshop training in prolonged exposure therapy improved provider self-efficacy, increased provider use of prolonged exposure, and reduced patients’ PTSD symptom severity.
- Post-workshop consultation appeared to be important not only for shifting provider attitudes and behavior, but also for improving patient outcomes, which is the ultimate goal of provider training.
- The promising impact of augmenting a provider workshop with expert case consultation may justify this relatively resource intensive effort.

## Background

Despite the robust evidence supporting the efficacy of prolonged exposure (PE) for posttraumatic stress disorder (PTSD) [1], PE is underutilized [2–4]. This underutilization is consistent with the low use of exposure therapy [5] and evidence-based psychotherapies (EBPs) in general [6, 7], highlighting the need for research on strategies to improve EBP implementation. One barrier that impedes EBP use is lack of therapist skill in delivering the treatment [8]. Indeed, Becker et al. [9] found that few providers who treat PTSD were trained in PE, and inadequate training was the most common reason for not using PE.

The US Departments of Defense (DoD) and Veterans Affairs (VA) have each pursued large-scale dissemination-implementation initiatives to increase the use of PE for PTSD, with differential success. In the VA PE roll-out, training included 4-day workshops followed by individual weekly consultation with 2 patients. An uncontrolled evaluation of this model found that consultation was associated with improved provider attitudes toward PE [10], which is independently associated with subsequent therapy adoption [11]. Eighteen months after PE consultation, 71% of VA providers reported using PE [12]. In contrast, the DoD adopted a workshop-only PE training model. Only 3% of DoD behavioral health providers listed PE as one of their primary treatments for patients with PTSD [13]. Consistent with broader implementation research findings, this pattern suggests that therapists require support after initial training in order to adopt the treatment (see [14] for a review). Consultation may also impact patient outcomes. For example, Monson et al. [15] found that consultation in cognitive processing therapy (CPT), including discussion of cases and conceptualization without audio review, resulted in significantly greater reductions in patients’ PTSD outcomes relative to no consultation in a VA setting. This study also found that audio review was not a necessary component for consultation to improve patient outcomes. Several other uncontrolled studies have found similar effects [16–18]. However, to our knowledge, prior PE implementation studies evaluating post-workshop consultation have not examined provider behavior and patient outcomes. This is an important gap in the literature, as therapists sometimes express reluctance to utilize exposure therapy, particularly in the presence of comorbid conditions [19]. Furthermore, whereas Monson et al.’s [15] study was conducted in the VA, there may be important organizational factors within the military that alter the impact of consultation on provider and patient outcomes in this setting. We are not aware of any controlled trials of consultation as an implementation strategy for improving provider and patient outcomes within military clinics.

Relative to workshops alone, case consultation requires significant time and financial resources for both the providers and consultants. Therefore, it is important to determine the incremental utility of this implementation strategy, both on PE use and on patient outcomes in large systems such as the military. This study used a hybrid type III effectiveness-implementation design, in which the primary goal was to determine the utility of an implementation strategy and the secondary goal was to assess clinical outcomes associated with the strategy [20]. The current study evaluated the impact of post-workshop consultation over 6–8 months on provider self-efficacy, expectations for patient improvement, beliefs related to PE, use of PE, and patients’ outcomes. We hypothesized that, compared to workshop alone (“Standard” training), the addition of post-workshop consultation (“Extended” training) would (1) increase provider self-efficacy for delivering PE, expectations for patient improvement following PE, and positive beliefs about PE; (2) increase the use of PE to treat patients with PTSD; and (3) significantly reduce PTSD symptoms.

## Methods

The Madigan Army Medical Center Institutional Review Board (IRB) served as the IRB for the three military sites. The IRBs at the University of Pennsylvania and the University of Texas Health Science Center at San Antonio as well as the US Army Human Research Protections Office also approved the protocol. Providers and patients who participated provided written informed consent.

### Providers

Providers were active duty or civilian behavioral health providers (*N* = 103) working at behavioral health clinics at three US Army medical treatment facilities: Evans Army Community Hospital at Fort Carson, Colorado; William Beaumont Army Medical Center at Fort Bliss, Texas; and Blanchfield Army Community Hospital at Fort Campbell, Kentucky. Providers met inclusion criteria if their responsibilities included delivering individual psychotherapy to adult patients and more than 20% of patients in their caseload had trauma-related difficulties. Providers were excluded if they planned to terminate their position within the next year or if they had extensive training in PE, defined as previous participation in a 4-day PE training workshop and use of PE with > 3 PTSD patients in the past year.

### Patients

Patients were active military personnel with PTSD symptoms (*N* = 242) who were receiving or seeking individual psychotherapy from a participating provider. The inclusion criterion was that they have clinically significant PTSD symptoms (score of ≥ 25 [21] on the Clinician-Administered PTSD Scale for *DSM-5* [CAPS-5] [22]). Exclusion criteria were current bipolar disorder I or psychotic disorder, evidence of a moderate or severe traumatic brain injury, or current suicidal ideation severe enough to warrant immediate attention.

### Procedures

The study design was a 2-armed randomized implementation trial comparing two PE training models: Standard training (workshop only) and Extended training (workshop plus consultation). Behavioral health providers were invited to participate in a 4-day PE workshop conducted on post. Initial PE workshops at each site were followed by additional on-site workshops as new providers enrolled in the study. Interested providers then completed informed consent with research staff. Local leadership agreed to support changes to participating providers’ schedule templates to allow providers to have 2 timeslots of 90 min each week during the study. Providers at each site were randomized 1:1 by the study coordinators using an online random-number generator. Unfortunately, the random-number method for treatment assignment resulted in more providers being assigned to the Standard condition (*n* = 60) than to the Extended condition (*n* = 43). Unbalanced randomization can occur naturally (though infrequently) when using pure randomization methods. Fortunately, as reported later in the “Results” section, this uneven assignment did not result in treatment condition differences between providers on any of the baseline demographic or study variables. Data were collected from January 2014 to January 2018, when target enrollment was met, and the final data point was collected. After the workshop, those randomized to the Extended condition received weekly individual telephone consultation from a PE expert, including the review of session videotapes, with a goal of 2 PE training cases over 6–8 months. Providers obtained consent from patients who were PE training cases to video-record the sessions for the purpose of consultation. PE training cases were not invited to participate in the study. Sessions of patients who were study participants were *not* video-recorded.

Providers were asked to complete online surveys prior to the workshop, immediately after the workshop, and at 3-month intervals for up to 18 months after finishing training. However, since one purpose of this study was to train as many providers as possible, providers were trained up until about 9 months before completion of the study. So, some providers who enrolled in the study only had time to provide a few (as little as 2) assessments of attitudes before the study ended. During the training phase, providers in the Extended condition were encouraged to implement PE with at least two training cases. During the data collection phase, providers were encouraged to use their clinical judgment when deciding which clinical issue to focus on and which treatment approach to use, allowing for an evaluation of the impact of the implementation strategy (Standard versus Extended) on providers’ selection of treatment approaches. These instructions aimed at eliminating the bias of demand characteristics that might influence greater use of PE while providers were in the study. Providers completed a checklist of the treatment procedures used with patients after each psychotherapy session.

Patients with significant PTSD symptoms (PTSD Checklist for *DSM-5* [PCL-5] ≥ 25 [23] [completed as part of routine care]) receiving treatment from participant-providers were invited by their provider to provide data for the study. Interested patients met with research staff to provide informed consent. They then met with an independent evaluator (IE), blind to the type of therapy the patient received and to the type of training received by the provider, who assessed their PTSD symptoms on two occasions: at study enrollment and after 8–15 sessions or 5 months, whichever came first. The second assessment occurred from sessions 3–15 (*M =* 9.4 for Standard patients, *M* = 9.1 for Extended), with variability due to factors such as patient transfer to another military installation, deployment, and inability to continue treatment. IEs provided a summary of the assessment findings to the patient’s provider. The study protocol and the statistical analysis plan are included in the online supplement.

### Provider measures

#### Provider demographics

This form assessed demographics and professional information (see Table 1).

**Table 1:** Provider demographic characteristics and training background

	Standard (*n* = 60)	Extended (*n* = 43)
**Demographic Characteristic**^**a**^		
Gender, No. (%)		
Male	20 (33.3)	18 (41.9)
Female	40 (66.7)	25 (58.1)
Ethnicity, No. (%)		
Hispanic	7 (11.7)	6 (14.0)
Non-Hispanic	53 (88.3)	37 (86.0)
Race, No. (%)		
Asian	3 (5.0)	1 (2.3)
Black	6 (10.0)	6 (14.0)
White	46 (76.7)	33 (76.7)
Other	5 (8.3)	3 (7.0)
Marital status, No. (%)		
Never married	0 (0.0)	1 (2.3)
Relationship, not cohabitating	3 (5.0)	3 (7.0)
Relationship, cohabitating	4 (6.7)	0 (0.0)
Married	41 (68.3)	26 (60.5)
Separated or divorced	10 (16.7)	13 (30.2)
Widowed	2 (3.3)	0 (0.0)
Education, No. (%)		
Master’s degree	37 (61.7)	29 (67.4)
Doctoral degree	23 (38.3)	14 (32.6)
Length working for the military, mean (SD), years	8.46 (7.16)	9.42 (6.89)
Current employment status, No. (%)		
GS civilian	42 (70.0)	31 (72.1)
Civilian contractor	1 (1.7)	2 (4.7)
Military	17 (28.3)	10 (23.3)
**Pretraining Background**		
Profession, No. (%)		
Psychologist	22 (36.7)	13 (30.2)
Social worker	35 (58.3)	28 (65.1)
Mental health counselor	1 (1.7)	0 (0)
Other	2 (3.3)	2 (4.7)
Type of clinic working at, No. (%)		
Outpatient PTSD clinic/PTSD clinical team	1 (1.7)	3 (7.0)
Outpatient mental health clinic	49 (81.7)	34 (79.1)
Primary care clinic	0 (0.0)	1 (2.3)
Outpatient addictions clinic	1 (1.7)	0 (0.0)
Women’s trauma program	1 (1.7)	0 (0.0)
Other	8 (13.3)	5 (11.6)
Working role, No. (%)		
Director of clinic	7 (11.7)	4 (9.3)
Assistant director of clinic	0 (0.0)	1 (2.3)
Full-time staff member	42 (70.0)	31 (72.1)
Part-time staff member	1 (1.7)	1 (2.3)
Other	10 (16.7)	6 (14.0)
Years of clinical experience, No. (%) (*n* = 102)		
Less than 1	4 (6.8)	1 (2.3)
1–5	11 (18.6)	13 (30.2)
6–10	13 (22.0)	9 (20.9)
11–15	4 (6.8)	7 (16.3)
16–20	8 (13.6)	6 (14.0)
20+	19 (32.2)	7 (16.3)
Years of clinical experience treating PTSD, No. (%)		
Less than 1	8 (13.3)	4 (9.3)
1–5	16 (26.7)	22 (51.2)
6–10	15 (25.0)	10 (23.3)
11–15	6 (10.0)	3 (7.0)
16–20	6 (10.0)	1 (2.3)
20+	9 (15.0)	3 (7.0)
Direct patient care hours completed in a week, mean (SD) (*n* = 94)	24.63 (7.53)	25.93 (13.57)
Primary theoretical orientation, No. (%)		
Psychodynamic/psychoanalytic	4 (6.7)	0 (0)
Cognitive	4 (6.7)	4 (9.3)
Behavioral	1 (1.7)	2 (4.7)
Cognitive-behavioral	33 (55.0)	25 (58.1)
Humanistic (existential, gestalt, Rogerian)	3 (5.0)	1 (2.3)
Interpersonal	1 (1.7)	1 (2.3)
Family systems	0 (0)	1 (2.3)
Eclectic/integrative	12 (20.0)	8 (18.6)
Other	2 (3.3)	1 (2.3)
Number of PTSD patients currently treating, No. (%)		
None	5 (8.3)	4 (9.3)
1–10	30 (50.0)	27 (62.8)
11–20	17 (28.3)	11 (25.8)
21–30	3 (5.0)	1 (2.3)
31–40	4 (6.7)	0 (0)
41–50	1 (1.7)	0 (0)
Number of PTSD patients treated in last 6 months, mean (SD)	15.00 (16.72)	15.42 (21.15)

*GS* general schedule, *No*. number, *PE* prolonged exposure therapy, *PTSD* posttraumatic stress disorder

^a^Age was not collected in accordance with our regulatory approval

#### Provider measure of attitude

This 52-item measure inquired about treatment practices, attitudes, expectancies, and beliefs. It included 8 items assessing self-efficacy in delivering PE (e.g., I can effectively guide patients through imaginal exposure), 1 item assessing expectations for patient improvement following PE (How effective do you think PE, if delivered competently, would be at improving your patients’ PTSD symptoms and functioning?), 21 items assessing “beliefs about PE” (e.g., Asking patients to discuss traumatic memories in PE may retraumatize them) using the Therapist Beliefs about Exposure Scale [24] modified to refer specifically to PE, 7 items assessing the proportion of PTSD patients with whom various psychotherapy practices are used (e.g., Provide a detailed rationale of how a treatment works), and 15 items assessing the degree to which various patient factors (e.g., Stabilized comorbid bipolar disorder; Anger; Dissociation) would deter providers from using PE. Self-efficacy items were scored 1 = *not at all confident* to 7 = *completely confident*. Expectations for patient improvement following PE were scored 1 = *not at all effective* to 7 = *very effective*. Beliefs about PE were scored 1 = *disagree strongly* to 5 = *agree strongly*. The internal consistencies of the self-efficacy measure and the beliefs measures in the present sample were *α* = .918 and *α* = .924, respectively.

#### Procedures used in treatment checklist

This form assessed the use of 14 different procedures in the previous treatment session, including an “other” option. Providers could check multiple procedures, including PE-related procedures (imaginal exposure, in vivo exposure, processing of traumatic memory, and providing rationale for PE) and non-PE procedures (e.g., supportive therapy, eye-movement desensitization and reprocessing, psychodynamic therapy).

### Patient measures

#### Patient demographics

This form assessed standard demographics (see Table 2).

**Table 2:** Patient demographic characteristics

	Standard (*n* = 172)	Extended (*n* = 70)
Gender, No. (%)		
Male	139 (80.8)	65 (92.9)
Female	33 (19.2)	5 (7.1)
Ethnicity, No. (%)		
Hispanic or Latino	40 (23.3)	13 (18.6)
Not Hispanic or Latino	132 (76.7)	57 (81.4)
Race, No. (%) (*n* = 241)		
American Indian/Alaskan Native	3 (1.7)	1 (1.4)
Asian	7 (4.1)	1 (1.4)
Native Hawaiian or other Pacific Islander	4 (2.3)	2 (2.9)
Black or African American	24 (14.0)	14 (20.3)
White	118 (68.6)	46 (66.7)
Others	16 (9.3)	5 (7.2)
Marital status, No. (%)		
Never married	11 (6.4)	2 (2.9)
Relationship, not cohabitating	13 (7.6)	2 (2.9)
Relationship, cohabitating	5 (2.9)	3 (4.3)
Married	113 (65.7)	49 (70.0)
Separated or divorced	30 (17.4)	14 (20.0)
Education, No. (%)		
Some high school	2 (1.2)	0 (0.0)
General education diploma	5 (2.9)	1 (1.4)
High school diploma	37 (21.5)	14 (20.0)
Some college	85 (49.4)	34 (48.6)
Associate degree	17 (9.9)	7 (10.0)
4-year college degree	19 (11.0)	13 (18.6)
Master’s degree	6 (3.5)	1 (1.4)
Doctoral degree	1 (0.6)	0 (0.0)
Military grade, No. (%)		
Enlisted		
E-1 to E-3	6 (3.6)	3 (4.3)
E-4 to E-6	122 (73.1)	43 (62.3)
E-7 to E-9	27 (16.2)	17 (24.6)
Warrant officer	6 (3.6)	1 (1.4)
Officer	6 (3.6)	5 (7.2)
Deployed in support of Operation Iraqi Freedom (OIF), No. (%) (*n* = 149)	86 (83.5)	42 (91.3)
Deployed in support of Operation Enduring Freedom (OEF), No. (%) (*n* = 188)	117 (90.7)	56 (94.9)
Deployed in support of Operation New Dawn (OND), No. (%) (*n* = 78)	29 (54.7)	13 (52.0)
Number of times deployed, No. (%) (*n* = 238)		
1	39 (23.1)	17 (24.6)
2	41 (24.3)	18 (26.1)
3	27 (16.0)	9 (13.0)
4	38 (22.5)	21 (30.4)
N/A	24 (14.2)	4 (5.8)
Typical duty during deployments, No. (%) (*n* = 237)		
Combat arms (i.e., infantry, etc.)	65 (38.7)	29 (42.0)
Combat support (i.e., engineer, etc.)	29 (17.3)	9 (13.0)
Combat service support (i.e., finance, chaplain, etc.)	52 (31.0)	27 (39.1)
N/A	22 (13.1)	4 (5.8)

*E-1 to E-3* junior enlisted, *E-4 to E-6* junior noncommissioned officers, *E-7 to E-9* senior noncommissioned officers, *N/A* not applicable, *No*. number

#### Clinician-administered PTSD scale for *DSM-5* (CAPS-5)

The CAPS-5 [22] is a structured interview that was used to assess PTSD symptom severity based on the *Diagnostic and Statistical Manual of Mental Disorders* (*DSM*-*5*). The CAPS-5 was administered by an IE blind to treatment condition at both patient assessment points (Cronbach’s *α* = .915).

### Interventions

#### Four-day PE workshop

Four-day PE workshops were conducted at each military installation throughout the duration of the study in order to continue to recruit providers throughout the course of the study. Workshops were conducted by PE experts from the University of Pennsylvania’s Center for the Treatment and Study of Anxiety (CTSA). The PE experts conducting the workshop were trained by the first author (E.B.F), the developer of PE. Each workshop was delivered over 4 consecutive days and included the following: (1) didactics on theoretical and empirical foundations of PE, (2) overview of the PE protocol, (3) instruction in individual therapy procedures with video-recorded examples, (4) monitored role-play practice with feedback, and (5) questions/discussion. Workshop participants received training materials and the PE treatment manual.

#### Post-workshop consultation

Consultation (for Extended condition providers) involved weekly 1-h telephone consultation from a PE expert with the goal of following 2 PE training cases. PE experts were certified PE providers and PE supervisors trained by E.B.F. Videos of the training cases were mailed to the CTSA and reviewed by the PE expert prior to the consultation call. Consultation calls included feedback on PE session recordings and/or discussion of PE training videos when providers did not have a training case to discuss. Twelve consultation phone calls were completed before the provider began inviting study patients.

### Data analytic plan

First, treatment condition differences on both provider and patient baseline characteristics were examined. Since there were 26 different characteristics compared, inflation of type I error was controlled using the Benjamini-Hochberg test. Multilevel models (MLMs) and generalized linear mixed models (GLMMs) were used to analyze the data. These intent-to-treat models included all providers and participants who provided at least 1 assessment. Per protocol, baseline level of outcome was used as a covariate when available. *P* was set at .05, two-sided.

For hypothesis 1, we expected greater improvement in PE attitudes (self-efficacy, expectancy, and beliefs about PE) over time in Extended vs. Standard providers. Since these variables improved initially and then leveled out, we used a quadratic growth curve to model change over time. The quadratic model fit the data better than other curvilinear models (e.g., log, hyperbolic, etc.) according to Akaike Information Criterion (AIC) and the Bayes Information Criterion (BIC).

For hypothesis 2 (provider use of PE components), we first examined the total number of PE components used by each provider across all their patients and sessions using a Poisson regression. Since some providers had more patients enrolled in the study than others, the total number of treatment sessions differed between providers, so number of treatment sessions was included as an “offset” in the analysis, converting the result to number of PE components used per session. In addition, we also compared the proportion of patients in each condition who were treated with any PE (defined conservatively as at least one session that included both imaginal and in vivo exposure). Patients were coded 1 (treated with PE) or 0 (not treated with PE) in a GLMM analysis (patients nested within providers). Since there were two outcomes for this hypothesis, *P* values were corrected for inflation of Type 1 error using the Benjamini-Hochberg correction.

The analysis for hypothesis 3 had three levels (the two repeated measurements of PTSD severity [baseline and final assessment] were nested within patients, who were nested within providers). Per our protocol, the primary outcome was PTSD symptoms at the second assessment. Since symptoms were expected to be lower for patients whose second assessment was after more sessions, session number of the final assessment was included as a moderator of treatment condition differences. And because the effect of sessions leveled out over time (i.e., the decrease in PTSD severity leveled out over sessions), “sessions” was coded as the square root of session number (this coding provided the best fit [lowest AIC and BIC] for the data). Follow-up GLMM analyses examined the secondary outcomes (change in PTSD diagnosis and clinically significant change, both dichotomous outcomes) using similar models. *P* values for the secondary outcomes were corrected for inflation of type 1 error using the Benjamini-Hochberg correction.

Initial analyses controlled for site as a covariate and moderator. However, site was not a significant factor in any of the analyses. Therefore, site was dropped and the analyses recomputed.

A priori power analyses, using RMASS2, indicated > .85 power to detect a medium effect size (*d* = .5) for hypothesis 1 (treatment condition differences in self-efficacy, expectancy, and beliefs toward PE) assuming 100 providers with at least 3 assessments each, thus setting sample size at *N* = 100. Post hoc power analyses, using actual *N* in each analysis, indicated that for hypothesis 1, power was > .90 power to detect a medium effect size. For hypotheses 2 and 3, the power to detect medium effects was > .80.

## Results

**Fig. 1:**
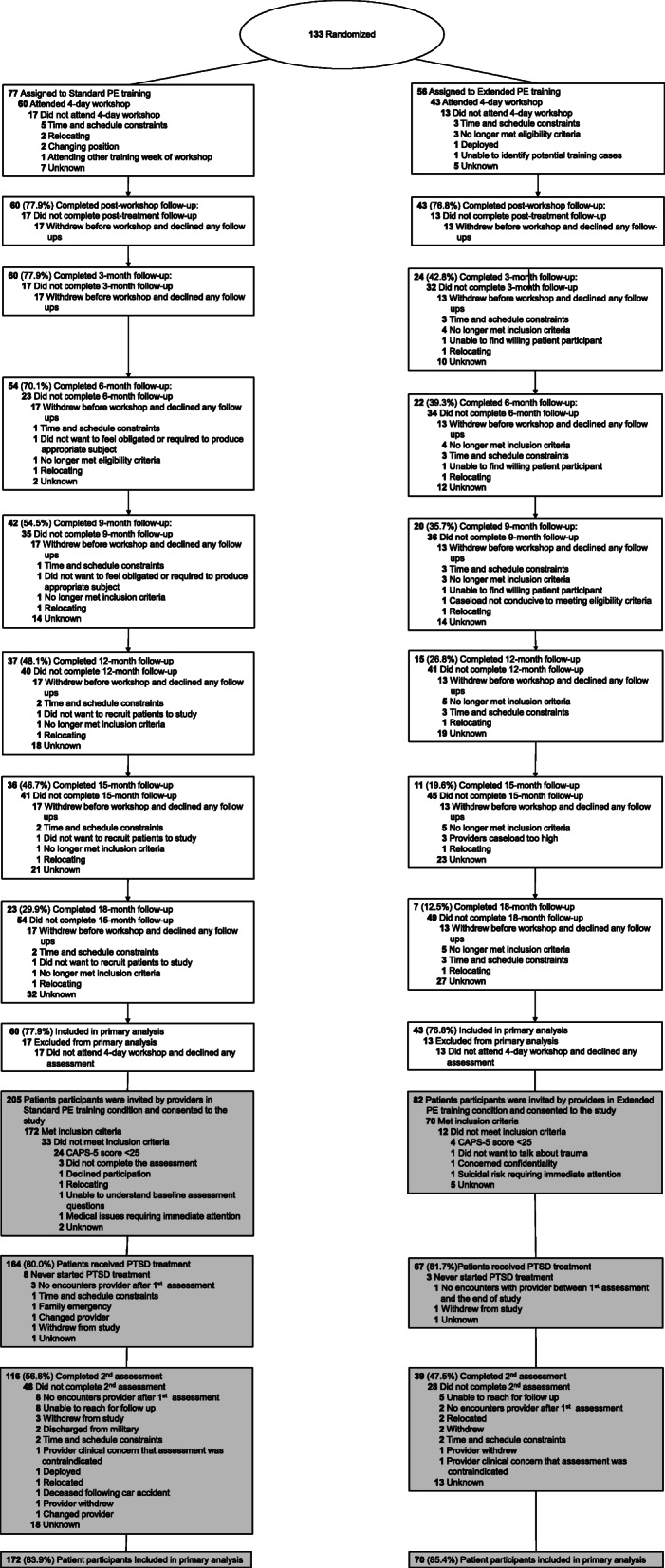
Diagram of provider participant flow

Figure 1 displays participant flow through the study. Provider and patient demographics and assessment information are reported in Tables 1 and 2, respectively. Out of the 26 variables reported in these tables, no treatment condition differences emerged after Benjamini-Hochberg correction. Substantive (but not significant) differences between conditions did exist on patient gender and “any comorbid anxiety disorder” (Extended condition females = 19.2%, comorbid anxiety disorders = 56.4%, Standard condition females = 7.1%, anxiety disorders = 28.6%). However, neither patient gender nor patient comorbid anxiety disorders was a significant moderator or covariate in the analyses of the patients, nor did including them in these analyses change any of the reported results.

### Differences in provider attitudes toward PE

**Fig. 2:**
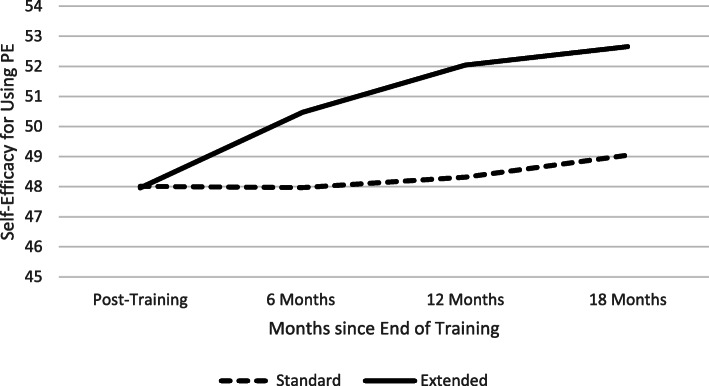
Months since end of training

For hypothesis 1, the number of assessments of PE attitudes per provider ranged from 1 to 8 in both treatment groups (*M* = 5.49, *SD* = 2.14). Consistent with hypothesis 1, Extended condition providers (*N* = 43) reported greater improvements in self-efficacy for delivering PE over time than did Standard condition providers (*n* = 60), training × time interaction: *b* = .83, 95% CI [.38, 1.27], *t*(79) = 3.71, *p* = .001, and *d* = .63 (Fig. 2). Self-efficacy for delivering PE improved significantly over time for Extended condition providers, *b* = .94, 95% CI [.58, 1.30], *t*(81) = 5.22, *p* < .001, and *d* = 1.16, but not for Standard condition providers. By the fourth assessment of attitudes, corresponding to 6–8 months after training, Extended condition providers reported significantly greater self-efficacy than Standard condition providers, *b* = 2.40, 95% CI [.49, 4.22], *t*(94) = 2.64, *p* = .01, and *d* = .54. There were no significant differences between training conditions in expectations for patient improvement following PE nor for beliefs about PE.

### Use of PE therapy components

Due to the fact that new therapists entered the study throughout the study, not all therapists had patients with PTSD enter the study; many had no qualifying patients before the conclusion of the study. There were 49 Standard condition providers (81.7%) and 23 Extended condition providers (53.5%) who had patients in the study and were included in the analyses for hypotheses 2 and 3. The proportion of Extended condition providers without patient data was larger because they could not enroll patients until after case consultation was completed, which took ≥ 6 months. There were no significant differences on demographics or on beliefs about PE (self-efficacy, effectiveness, positive attitudes) between providers who enrolled patients in the study and those that did not.

Poisson regression analyses showed that Extended condition providers used more PE components per treatment session (*M* = .9/session) than Standard condition providers (*M* = .5/session), *b* = .54, 95% CI [.15, .93], *t*(68) = 2.70, *p* = .012 (corrected), and *d* = .68. Importantly, this effect was limited to PE components; no significant difference between conditions emerged in non-PE components used during treatment sessions (*M*_Extended_ = 1.25 vs. *M*_Standard_ = 1.19). Further, GLMM analysis showed that the proportion of patients of Extended providers who actually received PE (defined as at least 1 session that included both in vivo and imaginal exposure) was greater than the proportion of patients of Standard providers who received PE (44% vs. 27%), *b* = .76, *t*(233) = 2.53, *p* = .012 (corrected), OR = 2.13.

### Patient outcomes

For hypothesis 3, Standard condition providers had 172 patients (3.5 patients per provider) in the study. Extended condition providers completed consultation in time to have 70 patients (3.0 patients per provider). The small difference in patients per provider was primarily due to the consultation delay before Extended condition providers could take patients into the study. Thirty-six percent of patients did not complete the second PTSD assessment, in part due to transfers/deployment. These patients did not differ from those who completed both assessments on demographics or baseline PTSD severity.

**Fig. 3:**
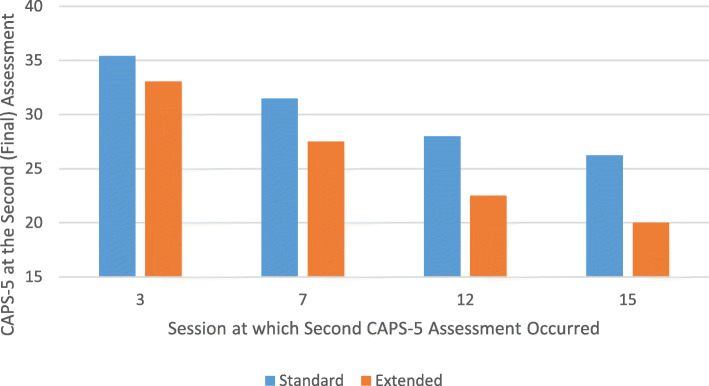
CAPS-5 for Patients at Their Second (Final) Assessment. Note: CAPS-5, Clinician Administered PTSD Scale for DSM-5. The session at which the second assessment occurred varied from session 3-15. This graph illustrates the treatment condition differences at 4 of those assessment points

Three-level MLM analyses included random effects for patients and for providers (which were non-significant) and baseline severity coded above/below median (this allowed inclusion of baseline CAPS in the MLM, providing intent-to-treat analyses). Improvement in PTSD symptoms per session was greater for patients of Extended condition providers than for patients of Standard providers, training × sessions interaction: *b* = − 1.81, 95% CI [− 3.57, − .04], *t*(263) = − 2.02, *p* = .045, and *d* = .62 (Fig. 3). For patients who completed 12 sessions, estimated PTSD symptoms were 28.0 for Standard condition patients vs. 22.5 for Extended condition patients, *b* = − 5.47, 95% CI [− 9.30, − 1.63], *t*(210) = − 2.81, *p* = .005, and *d* = .66.

Further analyses examined whether treatment condition differences were also apparent for the secondary outcomes: the loss of PTSD diagnosis and for clinically significant change (CSC) [25, 26]. These dichotomous outcomes were analyzed using GLMM with a logistic linking function. The independent variables in these models included training, sessions, and the training × sessions interaction as above. Using data from a large, multisite PTSD study [15] and using the Jacobson and Truax definition of CSC [25], CSC was defined as reliable change (1.96 × SD = 13) [26] plus having the outcome within the normal range (CAPS-5 < 20.89) [15]. Analyses indicated that the proportion of patients with loss of PTSD diagnosis was higher in patients of Extended condition providers compared to patients of Standard condition providers, *M*_extended_ = 76.9%, *M*_standard_ = 44.3%, *b*_difference_ = 1.20, 95% CI [.20, 2.20], *t*(391) = 2.37, and *p* = .019 (corrected). Similarly, the rate of CSC was significantly higher in patients of Extended providers compared to patients of Standard providers, *M*_extended_ = 50.2%, *M*_standard_ = 23.9%, *b*_difference_ = 1.16, 95% CI [.24, 2.08], *t*(21) = 2.63, and *p* = .019 (corrected).

Post hoc sensitivity analyses were conducted to control for the fact that Extended providers saw two more PTSD patients than Standard providers before treating PTSD patients for the study. To test whether this accounted for treatment condition differences, two sets of analyses controlled for number of PTSD patients treated by each provider during the study, one including, and one not including, the two consultation patients in the count of patients. Both sets of analyses showed that (1) number of PTSD patients treated was not related to patient outcomes either as a moderator or as a covariate and (2) patients of Extended providers improved more than patients of Standard providers even when analyses controlled for number of PTSD patients treated (either as a covariate or as a moderator). These results suggest that simply treating more PTSD patients does not seem to account for the superior outcomes for patients of Extended providers.

## Discussion

This hybrid type III effectiveness-implementation trial is the first study to demonstrate that consultation as an implementation strategy improved military providers’ self-efficacy for using EBP, use of EBP, and patients’ outcomes. These findings suggest that consultation is an effective implementation strategy for EBPs in the military health system and is also likely to be effective in other health systems.

Consistent with prior studies of post-workshop training [10], consultation increased provider self-efficacy in delivering PE, indicating that expert consultation improves confidence in providers’ ability to use PE effectively. Inconsistent with hypotheses, consultation did not increase expectations for improvement in PTSD following PE, or positive beliefs about PE, more than workshop training alone. A lack of effect of consultation on expectations for improvement is particularly interesting since it suggests that our results were not merely a result of expectations/self-fulfilling prophecies [27].

Consistent with hypothesis, consultation was associated with greater use of PE. The rate of use of PE components by Extended providers was almost 1 component (exactly .9 components) per treatment session, which was 80% greater than the use of PE components by Standard providers (.5 per treatment session). Further, Extended condition providers implemented PE (defined as at least 1 session with both in vivo and imaginal exposure) with a greater proportion of their PTSD patients. The rate of PE use was 50% higher among providers who received consultation compared to those who did not. This occurred despite the fact that providers were not directly incentivized to use PE in this study.

Also consistent with our hypothesis, consultation resulted in faster improvement and lower PTSD symptoms among patients. This study also found that patients of Extended providers were much more likely to lose their PTSD diagnosis than patients of Standard providers (77% compared to 44%). In addition, over twice as many patients of Extended providers demonstrated clinically significant change than did patients of Standard providers (50% vs. 24%). To our knowledge, this is the first study to demonstrate that PE consultation impacts not only providers but also the clinical outcomes of their patients. This finding is consistent with prior research in the context of veterans receiving CPT for PTSD [15] and with several uncontrolled studies [16–18]. This finding is important because improving patient outcomes is the ultimate purpose of provider EBP training, and hastening recovery is critical to minimizing patient suffering, cost of treatment, and achievement of military readiness efficiently.

These findings are consistent with a large body of evidence showing that workshop attendance alone is not sufficient to alter provider behavior, whereas follow-up consultation after the workshop can improve implementation [14]. This is the first study to our knowledge to demonstrate the benefit of consultation for improving implementation and patient outcomes.

Several limitations should be noted. First, to preserve external validity, we asked providers to self-report on the treatment procedures used in session, rather than using more intrusive data collection methods such as recording of sessions. It is possible that demand characteristics biased provider reporting, although providers were encouraged to use any treatment approach they felt was indicated for their patients. Second, many providers did not enroll any PTSD patients. Although providers reported having a caseload with least 20% having trauma-related difficulties, those who did not enroll patients to the study indicated that they either did not have patients with PTSD who were seeking psychotherapy (e.g., they may have presented for an evaluation or for crisis management) or did not have PTSD patients who met study criteria and agreed to participate. Importantly, there were no significant differences between providers who had study patients and those who did not on any of the assessed measures (on attitudes or on the 16 demographics). Third, relying on providers to invite patients may have biased the patient-participant pool. Study staff informed the participating providers frequently about the patients on their case load who met the screening criteria and encouraged providers to recruit all their eligible patients. However, ultimately, the provider made the decision of who to recruit. This is a limitation inherent to a type III hybrid implementation design, which required minimal influence on providers’ selection of patients in order to obtain more generalizable observations about the effect of the implementation strategy on providers’ behavior. Finally, our initial random number method for randomizing providers to treatment condition resulted in more providers being assigned to the Standard condition than to the Extended condition. Although this was unfortunate, unbalanced randomization can occur naturally (though infrequently) when using pure randomization methods rather than block randomization. Since there were no significant differences between providers assigned to the two treatment conditions, there is no indication that this uneven treatment assignment reflected a bias in treatment assignment. Thus, it is unlikely to be responsible for our obtained pattern of results. Finally, the findings may not generalize outside the military health system, as systems vary with regard to their organization, training and supervision practices, quality assurance practices, and the patient groups they serve.

## Conclusions

The results of this study demonstrate the importance of post-workshop consultation. Compared to Standard training, Extended training was associated with: (1) greater self-efficacy delivering PE, (2) greater use of PE and PE components, (3) faster improvement and lower PTSD symptoms among patients, (4) greater probability of loss of PTSD diagnosis among patients, and (5) more than twice the probability of experiencing clinically significant gains among patients. To our knowledge, this is the first study to demonstrate that PE consultation impacts not only providers but also the clinical outcomes of their patients. However, consultation remains costly. For example, in this study, sessions were video-recorded and mailed by providers to expert consultants who reviewed sessions and met with providers individually for 1 hour by phone weekly over several months. Recent evidence suggests that review of session recordings may not be necessary in effective consultation [15]. Future research could focus on critical components of consultation in order to provide benefits with lower cost.

## Supplementary information

**Additional file 1:.** Study protocol and the statistical analysis plan

## Abbreviations

AIC: Akaike Information Criterion
BIC: Bayes Information Criterion
CAPS-5: Clinician-Administered PTSD Scale for DSM-5
CSC: Clinically significant change
CTSA: University of Pennsylvania’s Center for the Treatment and Study of Anxiety
DoD: Department of Defense
DSM-5: Diagnostic and Statistical Manual of Mental Disorders, Fifth Edition
EBP: Evidence-based psychotherapy
GLMMs: Generalized linear mixed models
IRB: Institutional review board
MLMs: Multilevel models
PCL-5: PTSD Checklist for DSM-5
PE: Prolonged exposure therapy
PTSD: Posttraumatic stress disorder
VA: Department of Veterans Affairs
