# Supplementary material for: The effects of a prolonged exposure workshop with and without consultation on provider and patient outcomes: a randomized implementation trial
Source: Implement Sci. 2020 Jul 29;15:59. doi: 10.1186/s13012-020-01014-x (PMC7388467; doi:10.1186/s13012-020-01014-x)
Supplement: Supplementary file 1 — Additional file 1:. Study protocol and the statistical analysis plan [file 13012_2020_1014_MOESM1_ESM.docx]

**Protocol Number:** 818540

**Principal Investigator:** FOA, EDNA B

**Protocol Title:** Implementation of Prolonged Exposure in the Army: Is consultation necessary for effective dissemination?

**Short Title:** The Post-Traumatic Stress (PTS) Study

**Protocol Description:** This study will examine whether Prolonged Exposure (PE) for posttraumatic stress disorder (PTSD) can be successfully disseminated and implemented in the Army. 120 mental health providers will attend a 4-day PE workshop and will receive in-depth case consultation or no additional consultation following the workshop. Providers will complete surveys about their therapy practices. Patients seeking care for PTSD from providers will be recruited and consented to assess treatment response.

**1. Study Personnel**

*1.1. Principal Investigator*

Name: FOA, EDNA B

Dept. / School / Div.: 4419 - PS-Center for Treatment of Anxiety

Campus Address Mail Code: 6021

Address: 3535 Market St., Suite N600

City / State / Zip: PHILADELPHIA, PA 19104-3309

Phone: 215-746-3327

Fax: 215-746-3311

Pager:

Email: [foa@mail.med.upenn.edu](mailto:foa@mail.med.upenn.edu)

HS Training Completed: Yes

Training Expiration Date: 11/09/2017

Name of course completed: CITI Protection of Human Subjects Research Training - ORA

*1.2. Study Contacts*

Name: MCLEAN, CARMEN

Dept. / School / Div.: 4419 - PS-Center for Treatment of Anxiety

Campus Address Mail Code: 6021

Address: BLOCKLEY HALL, 423 GUARDIAN DR.

City / State / Zip: PHILADELPHIA, PA 19104-6021

Phone: 215-746-3327

Fax:

Pager:

Email: [mcleanca@mail.med.upenn.edu](mailto:mcleanca@mail.med.upenn.edu)

HS Training Completed: Yes

Training Expiration Date: 12/13/2018

Name of course completed: CITI Protection of Human Subjects Research Training - ORA

Name: BENHAMOU, KATHY

Dept. / School / Div.: 4419 - PS-Center for Treatment of Anxiety

Campus Address Mail Code: 6021

Address: BLOCKLEY HALL, 423 GUARDIAN DR.

City / State / Zip: PHILADELPHIA, PA 19104-6021

Phone: 215-746-3337

Fax:

Pager:

Email: [kathybe@mail.med.upenn.edu](mailto:kathybe@mail.med.upenn.edu)

HS Training Completed: Yes

Training Expiration Date: 06/08/2019

Name of course completed: CITI Protection of Human Subjects Research Training - ORA

*1.3. Other Investigator*

Name: MCLEAN, CARMEN

Dept. / School / Div.: 4419 - PS-Center for Treatment of Anxiety

Campus Address Mail Code: 6021

Address: BLOCKLEY HALL, 423 GUARDIAN DR.

City State Zip: PHILADELPHIA, PA 19104-6021

Phone: 215-746-3327

Fax:

Pager:

Email: [mcleanca@mail.med.upenn.edu](mailto:mcleanca@mail.med.upenn.edu)

HS Training Completed: Yes

Training Expiration Date: 12/13/2018

Name of course completed: CITI Protection of Human Subjects Research Training - ORA

**Responsible Org (Department/School/Division):**

4419 - PS-Center for Treatment of Anxiety

*1.4. Key Study Personnel*

Name: CAPALDI, SANDRA

Department/School/Division: PS-Center for Treatment of Anxiety

HS Training Completed: Yes

Training Expiration Date: 12/16/2017

Name of course completed: CITI Protection of Human Subjects Research Training - ORA

Name: YUSKO, DAVID

Department/School/Division: PS-Center for Treatment of Anxiety

HS Training Completed: Yes

Training Expiration Date: 01/08/2018

Name of course completed: CITI VA Human Subjects –Protection and Good Clinical Practices

Name: ZANDBERG, LAURA

Department/School/Division: PS-Center for Treatment of Anxiety

HS Training Completed: Yes

Training Expiration Date: 9/6/2018

Name of course completed: CITI Protection of Human Subjects Research Training - ORA

Name: ASNAANI, ANU

Department/School/Division: PS-Center for Treatment of Anxiety

HS Training Completed: Yes

Training Expiration Date: 9/11/2019

Name of course completed: CITI Protection of Human Subjects Research Training - ORA

Name: GALLAGHER, THEA

Department/School/Division: PS-Center for Treatment of Anxiety

HS Training Completed: Yes

Training Expiration Date: 03/15/2019

Name of course completed: CITI Protection of Human Subjects Research Training - ORA

Name: TURK-KARAN, ELIZABETH

Department/School/Division: PS-Center for Treatment of Anxiety

HS Training Completed: Yes

Training Expiration Date: 3/19/2020

Name of course completed: CITI Protection of Human Subjects Research Training - ORA

Name: BROWN, LILY

Department/School/Division: PS-Center for Treatment of Anxiety

HS Training Completed: Yes

Training Expiration Date: 8/16/2019

Name of course completed: CITI Protection of Human Subjects Research Training - ORA

**1.5. Disclosure of Significant Financial Interests***

*Does any person who is responsible for the design, conduct, or reporting of this research protocol have a FINANCIAL INTEREST?*

No

**Certification**

*I have reviewed the Financial Disclosure and Presumptively Prohibited Conflicts for Faculty Participating in Clinical Trials and the Financial Disclosure Policy for Research and Sponsored Projects with all persons who are responsible for the design, conduct, or reporting of this research; and all required Disclosures have been attached to this application.*

Yes

**2. Study Instruments**

*2.1. Provider Measures*

Provider Demographics & Background Survey: The Provider Demographics Form assesses standard demographics (ethnicity, gender, age, etc.) and professional information (e.g., military rank, type of schooling, etc.). It also includes items designed to assess providers training, theoretical orientation and experience providing evidence-based treatments, including their knowledge of PE, experience with PE, and attitudes towards PE. This measure will be completed by providers in both conditions.

Provider Measure of Attitudes: This is a 59-item measure of current treatment practices (7 items), beliefs about the effectiveness of PE (1 item), self-confidence in delivering PE (8 items), and perceptions about patient factors that would influence providers decision to use PE (22 items). In addition, this measure includes an embedded, modified version of the 21-item Therapist Beliefs about Exposure Scale (TBES; Deacon et al, 2013). All TBES items were modified to refer to PE specifically, rather than exposure therapy which is a more general term. The TBES has strong psychometric properties including excellent internal consistency ( = .90-.96), high test-retest reliability (r = .89; Deacon et al., 2013). The Provider Measure of Attitudes will be completed by providers in both conditions prior to attending the PE training workshop, after attending the PE training workshop, and at regular 3-month intervals over the course of 18 months following the completion of training (i.e. for providers in the standard condition, after the completion of the training workshop; for providers in the extended condition, after the completion of two supervised PE training cases).

Procedures Used in Treatment Checklist: This 4-item form is meant to collect data on what treatment techniques providers used during their sessions with study patients. Providers will also use the form to record patients’ biweekly PCL and PHQ-9 scores.

*2.2. Patient Measures*

Patient Demographics: The Patient Demographics Form assesses standard demographics (ethnicity, gender, age, etc.).

The Mini International Neuropsychiatric Interview (M.I.N.I.; Sheehan et al., 1998): The M.I.N.I. is a 30 minute diagnostic structured interview for 17 Diagnostic and Statistical Manual (DSM)-IV Axis I psychiatric disorders, including mood disorders, anxiety disorders, and substance use disorders. This instrument has been used in numerous studies of veteran samples (e.g., Kashdan, Frueh, Knapp, Hebert, & Magruder, 2006; Magruder et al., 2005; Stecker, Fortnery, Hamilton, & Ajzen, 2007). The complete M.I.N.I. will be administered and used to assess, in a standardized way, for evidence of any psychotic disorder that would exclude participants from treatment on this study.

The Clinician Administered PTSD Scale for DSM 5 (CAPS 5; Weathers, Blake, Schnurr, Kaloupek, Marx, & Keane, 2012): The CAPS is a 30-item structured interview that can be used to: 1) make current (past month diagnosis of PTSD; 2) make lifetime diagnosis of PTSD; and 3) assess PTSD symptoms over the past week. The CAPS interview yields a total symptom severity score and symptom cluster severity scores. In addition to assessing the 20 DSM-5 PTSD symptoms, questions target the onset and duration of symptoms, subjective distress, impact of symptoms on social and occupational functioning, improvement in symptoms since a previous CAPS administration, overall response validity, overall PTSD severity, and specifications for the dissociative subtype (depersonalization and derealization). For each symptom, standardized questions and probes are provided. The CAPS was designed to be administered by clinicians and clinical researchers who have a working knowledge of PTSD, but can also be administered by appropriately trained paraprofessionals. The CAPS takes 30-45 minutes to administer.

The CAPS assumes that an index trauma has already been identified by another means (e.g., by using a trauma checklist or structured interview questions) and does not include any questions to identify the index trauma. Thus, prior to administration of the CAPS, the assessor will ask the patient three questions in order to identify the index trauma. These three-questions are referred to as the Trauma Screen and will be administered at the first assessment only. At the second assessment, patients will complete a single question (Trauma Check) to confirm whether the previously identified index trauma was a major focus of his/her treatment.

Beck Scale for Suicidal Ideation (BSSI; Beck, Kovacs, & Weissman, 1979): The self-report version of the BSSI was will be used to evaluate the current intensity of the participants specific attitudes, behaviors, and plans to commit suicide. The BSSI has high internal consistency and strong concurrent validity with the clinician-administered version for both inpatients and outpatients (Beck, Brown, & Steer, 1997). This measure has been used in many studies of veteran populations (e.g., Bell & Nye, 2007; Cigrang et al., 2011; Koons et al., 2001). The original 21-item self-report requires that the patient fill out Part 2 (items 6-20) only if he/she previously endorsed items 4 or 5.

PTSD Checklist Civilian Version (PCL-C; Weathers et al., 1991): The PCL-C is a 17 item self-report measure that evaluates how much participants have been bothered by key PTSD symptoms in the past month. Patients will be asked to complete this measure, the PCL-5, or whichever PTSD measure is used as part of routine clinical care every two weeks, and their total score will be collected by the provider and listed on the Procedures Used in Treatment Checklist.

PTSD Checklist-5 (PCL-5; Weathers et al., 2013): The PCL-5 is a 20 item self-report measure that assesses/corresponds to the 20 DSM-V symptoms of PTSD, updated from the original PCL. Patients will be asked to complete this measure, the PCL-C, or whichever PTSD measure is used as part of routine clinical care every two weeks, and their total score will be collected by the provider and listed on the Procedures Used in Treatment Checklist.

Patient Health Questionnaire -9 (PHQ-9; Pfizer, 1999): The PHQ-9 is a commonly used self-report measure of depression that consists of 9 items that assess depressive symptoms. Each item is composed of four statements that reflect symptoms severity. The PHQ-9 has internal reliability of .89 and .86. It does correlate strongly with other measures of depression. (Huang, Chung, Kroenke, Deluded & Spitzer, 2005). The PHQ-9 has been used in numerous studies of military personnel. Patients will be asked to complete the PHQ-9 every two weeks, and their total score will be collected by the provider and listed on the Procedures Used in Treatment Checklist.

The State-Trait Anger Expression Inventory-2 (STAXI-2; Spielberger, 1999): The STAXI-2 is a 57-item inventory which measures the intensity of anger as an emotional state (State Anger) and the disposition to experience angry feelings as a personality trait (Trait Anger). The instrument consists of six scales measuring the intensity of anger and the disposition to experience angry feelings. Items consist of 4-point scales that assess intensity of anger at a particular moment and the frequency of anger experience, expression, and control. Only the State Anger subscale will be used in this study.

Alcohol Use Disorder Identification Test (AUDIT; Allen, Litten, Fertig, & Barbor, 1997): The AUDIT is a 10-item measure of at-risk alcohol use that has been widely used in studies of veterans (e.g., Bradley, McDonell, Kivlahan, Diehr, & Fihn, 1998; Bush, Kivlahan, McDonell, Fihn, & Bradley, 1998; Morton, Jones, & Manganaro, 1996). Items assess the quantity and frequency of drinking as well as the consequences associated with drinking. The maximum total score is 40, and a cut-off score of 8 or more has been shown to have a sensitivity and specificity of 96% in detecting at-risk alcohol use.

Credibility/ Expectancy Questionnaire Post-Treatment Survey (CEQ; Devilly & Borkovec, 2000): The CEQ Post-Treatment Survey is a 6-item measure of treatment expectancy and rationale credibility. Each item is rated on a 9- point scale (1= not at all, 9= extremely). The measure has demonstrated high internal consistency and good test-retest reliability (Devilly & Borkovec, 2000). The CEQ Post-Treatment Survey will be administered at the second assessment.

Client Satisfaction (CSQ; Larsen, Attkisson, Hargreaves, & Nguyen, 1979): This 8-item questionnaire measures satisfaction with treatment services received. Items are rated on a 4-point Likert scale, from 1 (excellent) to 4 (poor) with higher scores indicating less satisfaction. It has high internal consistency and is correlated with treatment completion, number of therapy sessions attended, and change in client-report symptoms (Attkisson et al., 1982). This will be given at the second assessment.

Additional Services Form (ASF): The Additional Services Form is a clinician-administered interview which includes 9 items in the First Assessment and 10 items in the Second Assessment. It assesses whether the patient had applied or intend to apply for military disability services. It also assesses patient participation in concurrent mental health services. This measure will be used to assess and control for additional mental health services or anticipated medical board review among patient-participants.

**2.3. Group Modifications**

There will not be any modifications to any study instruments for different groups. However, only the Extended PE training condition will complete the Provider Post-Consultation Measure of Attitudes because they will be the only group that receives consultation.

**2.4. Method for Assigning Subjects to Groups**

The study proposes to enroll approximately 100 participants in each condition. Participants will be randomized to conditions by a computer-based random list generator.

**2.5. Administration of Surveys and/or Process**

All participants will attend a 4-day workshop in prolonged exposure. All participants will all complete self-report measures at various time points: 1) prior to completing the workshop, 2) after completing the workshop, and 3) at 3-month intervals over the course of the 18 months following the completion of training (for standard providers, following the workshop; for extended providers, following their 2 supervised PE training cases). These self-report measures should take approximately 20-30 minutes to complete. Participants who are randomized to Extended PE training condition will additionally be asked to participate in weekly telephone consultation for two PE training cases, which we expect to last approximately 6 months. Patients of the participating providers who consent will be evaluated at two time points: once before they begin treatment (first assessment) and once after receiving 8-15 sessions of treatment, or after 5 months since the initial assessment, whichever comes first (second assessment). Patients who complete or leave the study before receiving 8 sessions of treatment will be asked to complete a second assessment at the time of termination. The consent and first assessment sessions will take a total of 2 to 2.5 hours. The second assessment will take approximately 1 hour. Patients will also be asked to complete self-report measures every 14 days. These measures can take 10-15 minutes to complete.

**2.6. Data Management**

Research files will be created for each study participant (provider and treatment-seeking PTSD patients). All data collection sheets will be coded. Research staff at each of the performance sites will assign a unique alphanumeric number to each participant and maintain the link between PHI and the code only at the local site. Research files will be maintained in a locked filing cabinet in a locked office that only study staff can access. From the data collection sheets, all coded research data will be entered into the study database that will be maintained by the University of Texas Health Science Center at San Antonio (UTHSCSA) using the same policies and procedures established for the South Texas Research Organizational Network Guiding Studies on Trauma and Resilience (STRONG STAR) PTSD Research Consortium. Access to the coded research data maintained by UTHSCSA will require a password protected login available only to authorized Biostatistics and Data Management Core staff. Data will be stored in a password-protected centralized database server on a Windows 2003 platform using Microsoft SQL 2005.

**2.7. Medical Information Disclosure***

*Does the research proposal involve the use and disclosure of research subject's medical information for research purposes?*

Yes

**If the answer is YES, indicate which items is provided with this submission:**

Modified research informed consent document that incorporates HIPAA requirements

**Primary Focus***

Sociobehavioral (i.e. observational or interventional)

**Protocol Interventions**

- Sociobehavioral (i.e. cognitive or behavioral therapy)

- Survey instrument

**3. Sponsors**

*3.1. Business Administrator*

Name: PRITCHETT, LAVINIA

Dept. / School / Div.: 4438 – PS-Psychiatry Business Office

Phone: 215-746-4439

Fax: 215-662-2238

Pager:

Email: [lbraxton@mail.med.upenn.edu](mailto:abbyd@mail.med.upenn.edu)

*3.2. Funding Sponsors*

Name: DEPARTMENT OF DEFENSE

Type: UPENN Federal

*3.3. Regulatory Sponsor*

Name: UNIVERSITY OF PENNSYLVANIA

Type: UPENN Internal

*3.4. IND Sponsor*

None

*3.5. Industry Sponsor*

None

**3.6. Project Funding***

Is this project funded by or associated with a grant or contract?

Yes

**Selected Proposals**

Proposal No. 10032353

**Title**

Implementation of Prolonged Exposure in the Army: Is Consultation Necessary for Effective Dissemination?

**Sponsor Funding**

*Is this study funded by an industry sponsor?*

No

**4. Multi-Site Research**

*4.1. Other Sites*

Site: William Beaumont Army Medical Center, Fort Bliss

Contact:

PI: MAJ Charles Zamora, LCSW

Mail: El Paso, TX 79920

Phone:

Email:

Site: Evans Army Community Hospital

Contact:

PI: Heather Campbell, Ph.D.

Mail: Fort Carson, CO 80913

Phone:

Email:

Site: Blanchfield Army Community Hospital

Contact:

PI: CPT Valerie Scott, Psy.D.

Mail: Fort Campbell, KY 42223

Phone:

Email:

**4.2. Management of Information for Multi-Center Research**

Reporting unanticipated problems involving risks to participants or others (UPIRSOs): The identified risk to both mental health providers and patients seeking treatment is the risk of breach of confidentiality. UPIRSOs will be submitted to the IRB within 10 working days of discovery, or as directed by the local IRB. There is no plan for an interim data analysis or report. Protocol modifications will be accomplished through amendments to the approved protocols at the local sites as well as the University of Pennsylvania. The Penn study staff will draft any needed modifications. Collaborators at the University of Texas Health Science Center San Antonio (UTHSCSA) will coordinate the submission of regulatory documents including protocol amendments to the military IRBs. The Penn study staff will submit regulatory documents including protocol amendments to the Penn IRB. Communication between Penn and the performance sites: The Penn investigators will hold regular teleconferences with the performance sites to monitor study progress, identify any adverse events and protocol deviations, communicate any changes or modifications to the study protocol, and problem solve with the site staff. Additionally, Penn investigators will make annual visits to the performance sites to conduct training and provide on-site consultation and monitoring.

**5. Protocol**

*5.1. Abstract*

This study will examine how evidence-based therapies (EBTs), such as Prolonged Exposure (PE) for posttraumatic stress disorder (PTSD) can be successfully disseminated and implemented in the Army by comparing two PE training models: Standard PE training (workshop only) and Extended PE training (workshop plus consultation). Two hundred (200) mental health providers at three medium-to large CONUS Army installations will be randomly assigned to either Standard PE training or Extended PE training. All providers will receive a 4-day PE workshop, along with the distribution of training materials and treatment manuals, conducted at the military site by the Overall Study PI, Dr. Edna Foa, and her team. Following the 4-day workshop, participants randomized to the Extended PE training will receive weekly phone consultation with a PE expert on two PE therapy cases followed by twice monthly telephone consultation as needed for six months. Participants randomized to the Standard PE training group will not receive any additional consultation following the workshop. Providers will not be required to use PE therapy to treat their patients with PTSD; using PE will be optional. A major outcome of this study is the proportion of the providers PTSD patients that are offered PE. The mental health outcomes of providers PTSD patients will also be monitored as a second major outcome. Patients seeking care for PTSD from participating providers will be recruited, consented and offered treatment for PTSD and related problems to prospectively and systematically assess what type of treatment they are offered and their response to treatment.

*5.2. Objectives*

5.2.1. Overall objectives

This study will examine how evidence-based therapies (EBTs), such as Prolonged Exposure (PE) for posttraumatic stress disorder (PTSD) can be successfully disseminated and implemented in the Army by comparing two PE training models: Standard PE training (workshop only) and Extended PE training (workshop plus consultation). We hypothesize that compared to Standard training, the Extended PE training will lead to: 1) Greater frequency of PE delivery; 2) Higher provider self-efficacy and positive attitudes towards PE; and 3) Superior PTSD patient outcomes. The study will answer the following questions: Does Extended PE training lead to significantly greater use of PE than does Standard PE training? Does Extended PE training lead to significantly greater changes in provider attitudes compared to Standard PE training? Does Extended PE training lead to significantly greater changes in PTSD patient outcomes versus Standard PE training?

5.2.2. Primary outcome variable(s)

The first set of Objectives concern provider behaviors, specifically hypothesizing that Extended Training will result in a greater proportion of PTSD patients treated with PE, use of PE with more complex patients (i.e., those with greater severity, complicating factors, and/or comorbidities), and better adherence to the PE protocol in those cases treated with PE. The measures to address this objective will be derived from self-report scales completed after consultation. When feasible, summary measures will be derived by summing items bearing on the same issue. For example, the Implementation Survey includes a 25-item section listing comorbid conditions and complicating factors. The fidelity analysis will be based on ratings by IEs of recorded sessions. Another set of Objectives concerns changes in provider attitudes. The outcome measures will be summary scores derived from the Implementation Surveys, using sums of relevant items as appropriate to operationalize provider self-efficacy, satisfaction and expectations for change.

5.2.3. Secondary outcome variable(s)

The third set of objectives concern changes in patient outcomes, specifically PTSD symptom severity, other symptoms, dropout, and satisfaction with treatment. These measures will be given at baseline and after 8-15 sessions of therapy, or after 5 months since initial assessment, whichever comes first.

*5.3. Background*

The Urgent Need for Effective PTSD Treatment in the Military

The current wars in Iraq and Afghanistan combined with wide implementation of mental health screening and management initiatives in the military have drastically increased the number of active duty soldiers who are identified as needing treatment for military-related trauma, and in particular, treatment for posttraumatic stress disorder (PTSD). An estimated 14% of Operation Iraqi Freedom/Operation Enduring Freedom/ Operation New Dawn (OIF/OEF/OND) military personnel meet criteria for PTSD (Tanelian & Jaycox, 2008). The greatly increased number of soldiers with PTSD has generated an enormous need to provide effective and efficient treatment for this disorder. This need, in turn, calls for a systematic effort to train military mental health providers in how to competently deliver short-term, efficacious, evidence-based treatments (EBTs) for PTSD. Treating PTSD is important not only to ameliorate PTSD symptoms, but also to prevent the devastating process associated with the chronicity of the disorder. Indeed, in the absence of effective treatment, PTSD frequently becomes chronic and comorbid with major depression, other anxiety disorders, substance use disorders (Kessler et al., 2005), poor physical health illnesses (Jakupcak, Luterek, Hunt, Conybeare, & McFall, 2008; Vasterling et al., 2008), and low quality of life (Zayfert et al.,2002).

Advantages of Prolonged Exposure Treatment for PTSD

Fortunately, we have developed highly effective and efficient treatments for PTSD. Among them are exposure therapies, cognitive therapies, and eye movement desensitization and reprcessing (EMDR). Exposure therapies, and in particular prolonged exposure (Foa, Hembree, & Rothbaum, 2007), possess the largest body of empirical evidence for their efficacy (IOM, 2007). Prolonged exposure (PE) is a specific exposure therapy program designed to help PTSD sufferers to emotionally process their traumatic experiences through repeated revisiting and recounting their trauma memories (imaginal exposure), and repeated, gradual confrontation with trauma-related, safe, situations that the person avoids (in vivo exposure). PE comprises three main components: (a) in vivo exposure to trauma reminders, typically as between-session assignments; (b) imaginal exposure to the memory of the traumatic event, both in session and between sessions; and (c) processing of imaginal exposure. Two additional less central components are: (d) psycho-education about the nature of trauma and (e) training in controlled breathing.

As noted above, among the evidence based treatments (EBTs) for PTSD, PE has gained the most empirical evidence for its efficacy. As a result, PE has been identified in the joint VA-Department of Defense Clinical Practice Guideline for PTSD (VA-DoD Clinical Practice Guideline Working Group, 2010) as strongly recommended for use with veterans with PTSD. The 2007 report issued by the Institute of Medicine (IOM) concluded that exposure therapy was the only treatment for PTSD with sufficient evidence for its efficacy. Numerous randomized controlled trials indicate that PE is effective in reducing PTSD symptoms (see Cahill, Rothbaum, Resick & Follette, 2009), and is associated with rapid change and maintenance of treatment gains over time (e.g., Foa et al., 2005; Powers, Halpern, Ferenschak, Gillihan, & Foa, 2010; Taylor et al., 2003). In addition to greatly reducing PTSD symptoms, PE also reduces depression, general anxiety, guilt, anger, and anxiety sensitivity, and improves social functioning and health (Keane, Marshall, & Taft, 2006; Rauch et al., 2010). Moreover, PE is effective in treating PTSD related to a wide range of traumas as well as PTSD in comorbid population, including traumatic brain injury (Rauch, unpublished), alcohol dependence (Foa et al., in preparation), borderline personality disorder (Harned et al., 2012), and major depression (Hagenaars, van Minnen, & Hoogduin, 2010).

Importantly, the efficacy of PE was studied by the greatest number of independent research groups around the world. Compared to other EBTs for PTSD, the techniques used in PE are relatively simple, the manual is highly structured, and the procedures are stream-lined, easy to learn, and straightforward to deliver. Furthermore, several studies have shown that patients prefer exposure therapy over other types of treatment. For example, PE is preferred over medication (among women exposed to trauma: Angelo, Miller, Zoellner, & Feeny, 2008; among women with PTSD: Feeny, Zoellner, Mavissakalian, & Roy-Byrne, 2008), and over other EBTs for PTSD (Becker, Darius, & Schaumberg, 2007). Thus, PE has many characteristics of the optimal candidate for widespread dissemination: it is effective with a wide range of PTSD sufferers, it is relatively easy to learn and deliver, and it is the preferred treatment type among patients.

The research findings and the clinical considerations discussed above place PE as an excellent candidate for dissemination in large systems such as the VA and the Army. There is a small pool of experts on treatment of PTSD related to military trauma and even fewer experts with experience in disseminating EBT within large government systems. Efforts to disseminate PE have been spearheaded by the developer of PE, Dr. Foa who has worked closely with the VA for more than a decade, and with the Army and the DoD for the past 3 years. Dr. Foa is a world leader in research on EBT for PTSD and has created a specific training model for PE and a team that is experienced in implementing it. These resources will be used in the proposed research.

The Challenge of Disseminating Evidence-Based Treatment

At present, most people who suffer from PTSD do not receive evidence-based treatment (e.g., Kessler, Sonnega, Bromet, Hughes, & Nelson, 1995; Kulka et al., 1990), primarily because the availability of professionals who practice EBTs for PTSD is woefully limited (e.g., Becker et al.,2004; van Minnen et al., 2010). Accordingly, despite the well-documented effectiveness of PE and its recommendation in numerous practice guidelines, relatively few mental health providers deliver PE (Rosen et al., 2004; van Minnen et al., 2010). This state of affairs is not unique to PE; studies have documented insufficient use of exposure therapy in the treatment of other anxiety disorders (Freiheit, Vye, Swan, & Cady, 2004), and low rates of EBT delivery in general (Goisman, Warshaw, & Keller, 1999; Jameson, Chambless, & Blank, 2009).

The primary reason for the large discrepancy between the high efficacy of PE and its low utilization for PTSD patients is lack of adequate training in PE or other EBTs. Becker et al., (2004) surveyed psychologists to examine the extent to which providers were trained in, and used PE with their PTSD patients. The vast majority of providers reported no or modest experience in treating PTSD, and only a small minority had extensive experience treating this disorder. A minority of the providers reported that they had some training in PE, and even fewer reporting utilizing PE. Inadequate training was the single largest reported cause of not using PE. It follows that in order to increase the use of PE, providers need to receive effective training.

There is growing body of research indicating that EBTs can be effectively disseminated. Since 1995, there have been systematic and extensive efforts to transport PE from academic centers to community clinics in Philadelphia (Foa et al., 2005) and abroad (e.g., Israel, Japan, China). The most ambitious project involved disseminating PE throughout the VA system in the US as part of the VA Central Office initiative to disseminate EBTs for several mental health disorders. The project has been quite successful: even before the implementation of a systematic plan to accommodate the use of PE in the VA, 45% of providers who were trained are using PE regularly with their PTSD patients (Karlin et al., 2010). The aim of this program was to provide the VA with sustainable capacity to train and supervise their mental health practitioners in the delivery of EBTs for PTSD without the need for ongoing outside instruction. In 2009 alone, a total of 968 clinicians were trained to provide PE within the VA system, and an additional 269 were trained outside of VA in coordination with the PE role out initiative. Thus, top-down dissemination strategies appear to be an effective way to train a large number of mental health professionals in a short period to deliver PE for PTSD. Other research on disseminating PE has shown that community-based clinicians can be trained to effectively implement PE for PTSD when provided with comprehensive training that includes expert consultation (Foa et al., 2005, Karlin et al., 2010).

How Can We Increase Providers Use of PE Most Effectively?

One feature of the VA training program that is thought to contribute considerably to the effectiveness of the dissemination is the implementation of comprehensive consultation (i.e., supervision) on two PTSD cases after a 4-day workshop on how to deliver PE. Indeed, a survey of VA providers found a significant increase in self-confidence in delivering PE and in anticipated benefits from PE, and a significant decrease in anticipated problems in delivering PE from post-workshop to post-consultation (Ruzek, Schnurr, Vasterling, & Friedman, 2011). Indeed, the rationale for including consultation on two cases as part of the training program is the hypothesis that the support and guidance provided by an expert PE consultant is critical in promoting the confidence providers need in order to adopt PE into their practice and to ensure that the skills and techniques learned during the workshop are implemented correctly. Evidence in support of this hypothesis comes from our own experience training US Army providers in PE, as well as from studies examining different training models for evidence-based treatments. Between 2007 and 2009, we provided 19 PE workshops to over 600 providers who treat active military personnel in the Army. In contrast to the PE training in the VA, the Army training plan did not require consultation following the 4-day workshop, and therefore systematic supervision was not provided. Subsequent efforts to engage previously trained providers in a refresher workshop and local consultant trainings prove futile. These efforts revealed that very few providers who participated in the PE workshops systematically utilize this treatment in their practice with their PTSD patients.

The Critical Role of Consultation in Changing Providers Use of PE

Research on disseminating other EBTs clearly suggests that supervision plays an important role in successfully implementing EBTs among mental health professionals. For example, studies of Motivational Interviewing (MI) for substance abuse have demonstrated that training via workshops alone yields limited increased proficiency in delivering MI (Baer et al., 2004; Miller & Mount, 2001), whereas the addition of case consultation increased provider MI proficiency over training via workshop only (West, DiLillo, Bursac, Gore, & Greene, 2007). In another study 140 licensed substance abuse providers were randomized to one of five training conditions, and assessed MI proficiency at pre-training, and for up to a year post-training. All training conditions were superior to the waitlist condition; however, those who received case consultation in addition to a workshop showed greater improvement than did those who received only a workshop. Furthermore, although those in the workshop-only condition showed immediate improvement in proficiency at post-training, there was a reversal of gains by the 4-month follow up, whereas those who received consultation maintained high levels of proficiency throughout the 1-year follow-up period (Miller, Yahne, Moyers, Martinez, & Pirritano, 2004). These results strongly suggest that post-workshop case consultation is critical in order to ensure adoption of newly learned skills to clinical practice.

Although workshops and one-time trainings may increase provider knowledge and behavior to some extent (Henggeler et al., 2008), such brief training does not appear to raise provider competence or treatment fidelity to recommended levels (Sholomskas et al., 2005), and any demonstrated gains may be temporary (Miller et al., 2004). These findings are extremely troubling because high levels of treatment fidelity and provider competence have been linked to superior patient outcomes for numerous EBTs (e.g., Hogue et al., 2008; Feeley, DeRubeis, & Gelfand, 1999; Schoenwald, Carter, Chapman, & Sheidow, 2008). Thus, any system that aims to improve patient outcomes by implementing EBTs will need to carefully attend to the training model it selects.

Extended training that includes case consultation requires a significantly greater investment of resources than does more standard training such as a one-time workshop. The advantages of a workshop-only training model are that providers can complete the training in a few days and it requires relative little investment (in terms of time and money) from providers and leadership. However, if the workshop-only training model does not effectively change provider behavior, then not only is the investment wasted, but more important, the goal of increasing access to effective and efficient treatment among soldiers suffering from PTSD will remain unrealized.

What Kind of Training is Needed to Maximize Providers’ Use of Prolonged Exposure?

It is imperative that EBTs for PTSD be disseminated in military mental health clinics to meet the growing demand for effective and efficient treatment for PTSD in a timely manner. Moreover, the training should be conducted in a manner that will ensure sustainability of implementation and maintenance of treatment quality and adherence. As noted above, there is considerable evidence to suggest that without additional case consultation, there is minimal impact of stand-alone training workshops on provider behavior. However, providing post-workshop case consultation is costly and is limited by the availability of experts to provide extended supervision. Thus, it is important to determine whether the Extended PE training (which includes a workshop + consultation on two cases) is superior to the Standard PE training (i.e., workshop without consultation, given that it is a more costly, resource-intensive approach. This proposal addresses the question of what training model optimizes the success of disseminating and implementing effectively the use of empirically supported treatments for PTSD in the Army by examining the outcome of two PE training models.

**6. Study Design**

**Phase***

Not applicable

*6.1. Design*

This is a randomized clinical trial comparing two methods of training mental health providers in Prolonged Exposure (PE) treatment for Posttraumatic Stress Disorder (PTSD). Two hundred (200) mental health providers at three medium-to-large CONUS Army installations will be randomly assigned to either Standard PE training or Extended PE training. All providers will receive a 4-day PE workshop, along with the distribution of training materials and treatment manuals, conducted at the military site by the Overall Study PI, Dr. Edna Foa, and her team. Following the 4-day workshop, participants randomized to the Extended PE training will receive weekly phone consultation with a PE expert on two PE therapy clinical cases followed by twice monthly telephone consultation as needed for six months. Participants randomized to the Standard PE training group will not receive any additional consultation following the workshop. Providers will not be required to use PE therapy to treat their patients with PTSD; using PE will be optional. A major outcome of this study is the proportion of the providers PTSD patients that are offered PE. The mental health outcomes of providers PTSD patients will also be monitored as a second major outcome. Patients seeking care for PTSD from participating providers will be recruited, consented and offered treatment for PTSD and related problems to prospectively and systematically assess what type of treatment they are offered and their response to treatment.

*6.2. Study duration*

The study is expected to start in May 2014 and end in May 2017. Subject recruitment and data collection will mostly occur in years 2, 3, and 4. Data analysis will occur in year 4.

*6.3. Resources necessary for human research protection*

Describe research staff and justify that the staff are adequate in number and qualifications to conduct the research. Describe how you will ensure that all staff assisting with the research are adequately informed about the protocol and their research related duties. Describe access to a population that would allow recruitment of the targeted number of subjects. If medical or psychological services as a consequence of the research, describe how the subject will be referred to those services. Describe your facilities and justify that the facilities are adequate. Verify that there is sufficient time to conduct and complete the research.

*6.4. Research Staff*

**Rank, Name, Corps:** Edna B. Foa PhD, Overall Study Principal Investigator

**Title, Service and Department:** Professor, Psychology in Psychiatry and Director, Center for the Treatment and Study of Anxiety

**Current Duty Station/Address:** University of Pennsylvania, 3535 Market Street, 6th Floor, Philadelphia, PA 19104

**Role and responsibilities:** Dr. Foa will be responsible for the conduct of all aspects of the research protocol, including initial submission, amendments, and required reviews; oversee all research activities; monitor subject enrollment, including progress in treatment and follow up compliance; responsible for guiding data analysis and publication submission after the conclusion of data collection.

**Rank, Name, Corps:** Carmen P. McLean, Ph.D, Co-Investigator

**Title, Service and Department:** Assistant Professor of Psychology in Psychiatry, Center for the Treatment and Study of Anxiety

**Current Duty Station/Address:** University of Pennsylvania, 3535 Market Street, 6th Floor, Philadelphia, PA 19104

**Role and responsibilities:** Dr. McLean will be responsible for the coordination of the project, including patient recruitment and ensuring a smooth flow of data throughout the life of the project, supervising research personnel including directly supervising the research assistant on the project, and traveling to the study sites to implement the proposed research. Dr. McLean also will participate in weekly conference calls between the study personnel and the site coordinators.

**Rank, Name, Corps:** Laurie Zandberg, Psy.D., Study Coordinator.

**Title, Service and Department:** Post-Doctoral Fellow, Center for the Treatment and Study of Anxiety.

**Current Duty Station/Address:** University of Pennsylvania, 3535 Market Street, 6th Floor, Philadelphia, PA 19104.

**Role and responsibilities:** Dr. Zandberg will work directly with Dr. McLean to help with coordination of the project, including patient recruitment and ensuring a smooth flow of data throughout the life of the project, supervising research personnel including directly supervising the research assistant on the project, and traveling to the study sites to implement the proposed research. Dr. Zandberg will also be responsible for coordinating with study consultants, and liaising between consultants, study BOAs, and study providers to ensure that consultation proceeds smoothly. Dr. Zandberg also will participate in weekly conference calls between the study personnel and the site coordinators.

**Rank, Name, Corps:** Alan Peterson, Ph.D., ABPP, Lt Col (ret), U. S. Air Force, Associate Investigator

**Title, Service and Department:** Professor of Psychology in Psychiatry, Division of Behavioral Medicine & Director STRONG STAR PTSD Research Consortium

**Current Duty Station/Address:** University of Texas Health Science Center at San Antonio, 7550 IH10 West, Suite 1325, San Antonio, TX 78229-5820

**Role and responsibilities:** Dr. Peterson will provide expert consultation regarding the implementation of this study at a military treatment facility, monitor conference calls, assist in meeting all military clinical requirements in the provision of PTSD treatment, assist in meeting all military regulatory requirements, coordinate with the STRONG STAR Data Core maintaining the study data, and assist with data analysis and interpretation.

**Rank, Name, Corps:** Sandra Capaldi, PsyD, Clinical Supervisor

**Title, Service and Department:** Clinical Supervisor, Center for the Treatment and Study of Anxiety

**Current Duty Station/Address:** University of Pennsylvania, 3535 Market Street, 6th Floor, Philadelphia, PA 19104

**Role and responsibilities:** Dr. Capaldi will supervise providers who receive the more intensive training experience and will assist with the Prolonged Exposure (PE) workshops for the clinicians. Dr. Capaldi has extensive experience in teaching and supervising PE. She will have access to study data including PHI.

**Rank, Name, Corps:** David Yusko, PsyD, Clinical Supervisor

**Title, Service and Department:** Clinical Supervisor, Center for the Treatment and Study of Anxiety

**Current Duty Station/Address:** University of Pennsylvania, 3535 Market Street, 6th Floor, Philadelphia, PA 19104

**Role and responsibilities:** Dr. Yusko will supervise providers who receive the more intensive training experience. Dr. Yusko has extensive experience in teaching and supervising PE. He will have access to study data including PHI.

**Rank, Name, Corps:** Thea Gallagher, PsyD, Clinical Supervisor

**Title, Service and Department:** Clinical Supervisor, Center for the Treatment and Study of Anxiety

**Current Duty Station/Address:** University of Pennsylvania, 3535 Market Street, 6th Floor, Philadelphia, PA 19104

**Role and responsibilities:** Dr. Gallagher will supervise providers who receive the more intensive training experience. Dr. Gallagher has extensive experience in teaching and supervising PE. She will have access to study data including PHI.

**Rank, Name, Corps:** Elizabeth Turk-Karan, PhD, Clinical Supervisor

**Title, Service and Department:** Clinical Supervisor, Center for the Treatment and Study of Anxiety

**Current Duty Station/Address:** University of Pennsylvania, 3535 Market Street, 6th Floor, Philadelphia, PA 19104

**Role and responsibilities:** Dr. Gallagher will supervise providers who receive the more intensive training experience. Dr. Gallagher has extensive experience in teaching and supervising PE. She will have access to study data including PHI.

**Rank, Name, Corps:** Lily Brown, PhD, Clinical Supervisor

**Title, Service and Department:** Clinical Supervisor, Center for the Treatment and Study of Anxiety

**Current Duty Station/Address:** University of Pennsylvania, 3535 Market Street, 6th Floor, Philadelphia, PA 19104

**Role and responsibilities:** Dr. Gallagher will supervise providers who receive the more intensive training experience. Dr. Gallagher has extensive experience in teaching and supervising PE. She will have access to study data including PHI.

**Rank, Name, Corps:** Anu Asnaani, PhD

**Title, Service and Department:** Assistant Professor, Center for the Treatment and Study of Anxiety

**Current Duty Station/Address:** University of Pennsylvania, 3535 Market Street, 6th Floor, Philadelphia, PA 19104

**Role and responsibilities:** Dr. Asnaani will join the study’s research team, and will travel to scheduled training workshops in order to assist with recruitment and retention efforts for provider-participants. She will have access to study data including PHI.

**Rank, Name, Corps:** Kathy Benhamou, BA, Research Assistant

**Title, Service and Department:** Research Assistant, Center for the Treatment and Study of Anxiety

**Current Duty Station/Address:** University of Pennsylvania, 3535 Market Street, 6th Floor, Philadelphia, PA 19104

**Role and responsibilities:** Ms. Benhamou will help coordinate all of the communication between the University of Pennsylvania and the four study sites. She will assist Dr. McLean in all aspects of project coordination. She will be responsible for keeping accurate logs of provider training, assisting in communication with the Institutional Review Boards, carrying out needed data entry, and preparing training materials. Ms. Benhamou will participate in the weekly telephone calls and will be responsible for coordinating study related travel for the study personnel. She will have access to study data including PHI.

Other Technical Staff, to include a Study Coordinator, BOA, and Research Assistant, will be hired by The Geneva Foundation. Their names, biographical information, and documentation of CITI training will be submitted to the IRB for review and approval prior to any interaction with study participants. Ensuring that all staff assisting with the research are adequately informed about the protocol and their research related duties. The Penn investigators will hold regular teleconferences with the performance sites to monitor study progress, identify any adverse events and protocol deviations, communicate any changes or modifications to the study protocol, and problem solve with the site staff. Additionally, Penn investigators will make annual visits to the performance sites to conduct training and provide on-site consultation and monitoring. Access to the Military Sites. In conjunction with her military funders, Dr. Foa and her team have made site visits to each of the military performance sites and secured their support for the conduct of this study.

*6.5. Penn Facilities*

Facilities at the Center for the Treatment and Study of Anxiety include 15 offices where patients are seen, all of which are equipped with digital video recorders, printers and computers. Also available are additional offices used for research related activities, including a laboratory equipped for medical screening and taking blood, conference rooms for group supervision, and double locked storage areas for securing confidential records and session tapes.

*6.6 Time to Conduct and Complete the Research.*

This project has funding for four years to conduct and complete the research according to the study timeline and milestone description (see Procedures section).

**7. Characteristics of the Study Population**

*7.1. Target population*

The primary group of participants will include male and female mental health providers regardless of racial or ethnic origin whose job duties include providing psychotherapy to service members with PTSD, and who can be reasonably certain that they will remain at the Army base for at least one year. The secondary group of participants will include male and female military beneficiaries regardless of racial and ethnic origin who are seeking treatment from the participating providers who exhibit clinically significant PTSD symptoms (defined as a severity score of 25 or greater on the Clinician-Administered PTSD Scale for DSM-5 (CAPS-5)).

**Subjects enrolled by Penn Researchers**

0

**Subjects enrolled by Collaborating Researchers**

120

*7.2. Accrual*

Provider-participants: The On-Site PI will present information about the study and participation to all mental health providers who provide psychotherapy at each site. Those who meet the inclusion and exclusion criteria will be invited to participate in the study and provided informed consent. The project coordinator and research assistant at each site will assist with the coordination of recruitment, screening, and informed consent of participants.

Patient-participants: Patients who screen positive for PTSD according to the PCL-5, PCL-C, or whichever screening measure is used as the performance site’s screening tool for PTS, and who are assigned to one of the provider-participants enrolled in the study will be invited to participate in the study and will meet with a member of the research team to review informed consent. Patients who consent to the study will complete a baseline evaluation with a BOA that includes the CAPS, the M.I.N.I, and self-report questionnaires.

*7.3. Key inclusion criteria*

Key inclusion criteria for provider-participants are the following:

- Male and female mental health providers whose job duties include providing psychotherapy to adult patients, ages 18-65, seeking treatment for PTSD.

- 20% of expected patient caseload with trauma related difficulties

- Support from local leadership to ensure that providers are granted the time and resources necessary to participate in study activities, including providing 90-minute once weekly sessions, time off to participate in the training workshop, time to participate in consultation , time to complete study assessments, etc.

Key inclusion criteria for patient-participants are the following:

- Male and female patients aged 18-65 who are seeking treatment from one of the mental health providers participating in this study.

- Clinically significant PTSD symptoms, defined as a severity score of 25 or greater on the Clinician-Administered PTSD Scale for DSM-5 (CAPS-5).

*7.4. Key exclusion criteria*

Key exclusion criteria for provider- participants are the following:

- Definite plans to terminate their position or relocate from the Army base at some point during the 1 year following training.

- Extensive previous training in Prolonged Exposure (PE), defined as participation in a 4-day PE training workshop at any point prior to the start of the study, and self-reported use of PE (including both imaginal and in vivo exposure techniques) to treat 4 or more patients with PTSD in the past year.

Key exclusion criteria for patient participants:

- Current bipolar disorder I or psychotic disorder (as determined by the MINI).

- Evidence of a moderate or severe traumatic brain injury (as determined by the inability to comprehend the baseline screening questionnaires).

- Current suicidal ideation severe enough to warrant immediate attention (as determined by the Beck Scale for Suicidal Ideation).

*7.5. Vulnerable Populations*

Children Form

Pregnant women (if the study procedures may affect the condition of the pregnant woman or fetus) Form

Fetuses and/or Neonates Form

Prisoners Form

Other

x None of the above populations are included in the research study

*7.6. Populations vulnerable to undue influence or coercion*

Study staff will emphasize to participating providers that the decision to implement PE in regular treatment of PTSD is voluntary and will not affect their status in the military or as a civilian or any benefits that they typically receive. Patients of participating providers will be informed that they can withdraw from the study at any time with no consequences and will be at no risk of losing their right to medical or psychological care with the providers.

*7.7. Subject recruitment*

Provider-participants: The On-Site PI will present information about the study to all mental health providers who provide psychotherapy. Mental health providers who meet the inclusion and exclusion criteria will be invited to participate in the study and provided informed consent. The project coordinator and research assistant at each site will assist with the coordination of recruitment, screening, and informed consent of provider participants.

Patient-participants: Patients seeking mental health services complete a packet of self-report measures via paper copy or the Behavioral Health Data Platform (BHDP) as part of the routine clinic intake process. In particular, patients will complete a self-report measure of PTSD symptom severity (the PCL-C, PCL-5, or whatever the performance site’s current screening tool for PTS is). Participating providers will be asked to notify patients who meet the screening criteria for PTS symptoms according to the screening measure administered of the option to participate in the study. Under a partial HIPAA waiver for screening, research staff will review the screening scores and alert participating providers of any patients they are scheduled to treat who meet the screening criteria). Research staff will track the names of referred patients that have been sent to providers using a tracking spreadsheet, which will be destroyed once recruitment goals are met. Provider-participants will inform the potential patient-participant that their participation is voluntary and will not affect their mental health care in any way. If the patient expresses interest in the study, the provider will sign the patient up for a meeting with the Behavioral Outcomes Assessor (BOA) via the AHLTA system when the patient checks out of the provider appointment. During the initial meeting with the BOA, the BOA will discuss the study details with the patient and consent the patient if he/she chooses to participate. Patients consenting will complete an intake assessment with the study Behavioral Outcomes Assessor (BOA).

**Subject compensation***

*Will subjects be financially compensated for their participation?*

No

*If there is subject compensation, provide the schedule for compensation per study visit or session and total amount for entire participation, either as text or separate document.*

Subjects will not be financially compensated for their participation.

**8. Study Procedures**

*8.1. Procedures*

This is a randomized clinical trial comparing two methods of training in Prolonged Exposure (PE) treatment. Consented mental health providers at three medium-to-large Continental United States (CONUS) Army installations will be randomly assigned to either implement Standard PE training or Extended PE training. Mental health providers at three military installations will be invited to participate in a 4-day PE workshop which will be conducted at the military site by PE experts from the University of Pennsylvania’s Center for the Treatment and Study of Anxiety. The PE expert conducting the workshop were trained by and work closely with Dr. Edna Foa, the developer of PE and one of the world’s leading experts in PTSD and PTSD treatment. As part of the PE workshop, the provider will receive training materials and treatment manuals. Following enrollment in the study, provider-participants will also receive an email/letter from the PI, Dr. Foa, thanking them for their study participation. Following the 4-day workshop, participants randomized to the Extended PE training will receive weekly case consultation from PE experts on two PE training cases including the review of video-tapes made of their two supervised cases. Patient consent to these video-recordings of clinical care will be accomplished by each sites local form for recording of patient care. These training case patients are not considered study participants. Provider will obtain consent from their patients to video-record using a separate clinical consent document. The video-recordings of the training cases will be copied onto DVD at the individual performance sites, labeled with the providers study number, and mailed directly to the University of Pennsylvania Center for the Treatment of Anxiety for review as part of case consultation teleconferences using an express service (e.g., FedEx) and tracked for receipt.

Participants randomized to the Standard PE training group will not receive any additional consultation following the workshop. In addition to mental health providers, patients seeking treatment from participating providers will be recruited and consented to examine their symptoms of PTSD and related problems during treatment. Providers will not be required to use PE therapy to treat their patients with PTSD; rather it will be an option for treatment. A diagnostic feedback form will be given to the provider by the BOA within 24-hours (with more time allotted as needed on a case-by-case basis) of completing both the first and second assessments and will contain a summary of the assessment data. This assessment data may be used by the provider, as he or she deems appropriate, for ongoing treatment decisions. Clinical care of patients enrolled in this study will not be video-taped. Choice of treatment modality will be one of the outcome measures; data on techniques used in treatment will be collected from study providers after every session with a study patient via the Procedures Used in Treatment Checklist. The Checklist will also be used by providers to record their study patients’ self-reported PTS and depression scores, which the providers will acquire from their AHLTA (medical record) session notes on a biweekly basis.

*8.2. Analysis Plan*

The first set of Objectives concern therapist behaviors, specifically hypothesizing that Extended Training will result in a greater proportion of PTSD patients treated with PE, use of PE with more complex patients (e.g. comorbidities). The analyses will be done using mixed effects regression models with repeated measures, incorporating as covariates baseline values on the outcome measures when available and confounded ancillary variables as needed. Planned between and within-group contrasts will be done at each assessment by t-test. The measures to address the first two objectives will be derived from self-report scales completed after consultation. When feasible, summary measures will be derived by summing items bearing on the same issue. For example, the Implementation Survey includes a 25-item section listing comorbid conditions and complicating factors. The fidelity analysis will be based on ratings by IEs of recorded sessions.

The second set of Objectives concerns changes in therapist attitudes. The analysis model will be mixed effects regression as above, with intervention arm the primary independent variable and repeated measures at post-consultation and at two and three years. The dependent measures will be summary scores derived from the Implementation Surveys, using sums of relevant items as appropriate to operationalize therapist self-efficacy, satisfaction and expectations for change. We anticipate that changes in therapist attitudes may appear very quickly in the training process, influenced by expectations about consultation and supervision at the earliest stages and experience of training as it is received.

The third set of Objectives concern changes in patient outcomes, specifically PTSD symptom severity, other symptoms, dropout, and satisfaction with treatment. The analysis design for continuous measures based on self-report scales is simple analysis of (co)variance on post-treatment outcomes (or equivalently, change), comparing intervention arms using pre-treatment measures as covariates. If dropout data are available at the individual patient level, simple chi-square, logistic regression or generalized mixed effects models will be used to compare interventions. For all patient level outcomes, mixed models may be needed because patients are nested within therapists. Preliminary analyses will explore the need to include therapist as a random design effect to account for possible clustering effects (Crits-Christoph & Mintz, 1991). For the therapist level outcomes, the impact of baseline patient (or therapist) characteristics that are confounded with intervention condition will be evaluated using analysis of covariance.

**Deception**

*Does your project use deception?*

No

*Are you conducting research outside of the United States?*

No

**9. Confidentiality**

**Data confidentiality**

x Paper-based records will be kept in a secure location and only be accessible to personnel involved in the study.

x Computer-based files will only be made available to personnel involved in the study through the use of access privileges and passwords.

x Prior to access to any study-related information, personnel will be required to sign statements agreeing to protect the security and confidentiality of identifiable information.

x Wherever feasible, identifiers will be removed from study-related information.

*9.1. Subject Confidentiality*

Research files will be created for each study participant (provider and treatment-seeking patients). All data collection sheets will be coded. Research staff at each of the performance sites will assign a unique alphanumeric number to each participant and maintain the link between PHI and the code only at the local site. Research files will be maintained in a locked filing cabinet in a locked office that only study staff can access. From the data collection sheets, all coded research data will be entered into the study database that will be maintained by the University of Texas Health Science Center at San Antonio (UTHSCSA) using the same policies and procedures established for the South Texas Research Organizational Network Guiding Studies on Trauma and Resilience (STRONG STAR) PTSD Research Consortium. Access to the coded research data maintained by UTHSCSA will require a password-protected login available only to authorized Biostatistics and Data Management Core staff. Data will be stored in a password-protected centralized database server on a Windows 2003 platform using Microsoft SQL 2005. Security of the Database. The project uses two Dell Servers, both with RAID configured multiple hard drives as well as a separate external daily backup. The physical layer of the internal network infrastructure is a Gigabit Ethernet backbone maintained by UTHSCSA System and Network Operations (SNO).

- Firewalls: The University provides a series of CISCO firewalls and switched routers to provide the first line of campus security. Additionally, an external agency is responsible for monitoring firewall activity and event logs to provide an independent analysis of intrusion attempts and trends. STRONGSTAR employs an additional firewall appliance maintained by SNO at the entrance of its virtual network to further protect its resources.

- Network Design: The University has subdivided its network into Virtual Local Area Networks (VLANs). A VLAN-based network is used to subdivide users and servers based on both physical and operational parameters. A user must be physically mapped into a VLAN before any of its resources may be accessed. The University has designated a single VLAN for STRONGSTAR-specific resources.

- Public Zone: The public zone is a networking environment in which the University firewall will allow standard Internet traffic (and special secure shell access) to enter. The STRONGSTAR web servers (not database servers) reside in the campus public zone. The web server is configured to limit its internal access to a single computer (over a specialized and heavily monitored port) within the STRONGSTAR VLAN.

- Virtual Private Networking: The University offers a Virtual Private Networking (VPN) client application to access basic university resources; however, the STRONGSTAR firewall has an independent VPN capability (via the CISCO firewall). Users outside the STRONGSTAR VLAN may access computer resources through this encrypted VPN access. Access to the STRONG STAR website is restricted to SSL (128 bit) encrypted connections only. If FTP or application-driven email is implemented we will implement encryption for those services as required. Authentication is by username and password. The password is hashed (one-way-encrypted) using MD5 encoding so it is nearly impossible to reverse engineer the hash and get a person's password. Password length is restricted to a minimum length of eight characters to make brute force hacking difficult. After three failures the user is locked out for a period of one hour, making brute force hacking difficult. Access is logged making hacking attempts easier to discover. Users have access to only what they need to see and do by using the least-privilege access security scheme. To protect this data, STRONG STAR system has developed the concept of functional group access augmented with traditional application role-based access.

- A functional group can be any logical grouping of people or users (e.g., research sites, departments, working groups, etc.). Each authorized user is matched to one or more functional groups. As subjects are registered into the system, they are in turn mapped to the same list of functional groups. In addition, since a subject may belong to multiple functional groups (e.g., registered on multiple protocols), each time a group registers a subject, the protocol specific information is also mapped to the users functional group. To increase the granularity of the data access, an application may implement roles specific to the customers’ needs. A data manager may need to view (and edit) all of a subjects protocol-specified research data, a protocol coordinator may need to view specific data items, have no need to edit research data, but have the requirement to enter information specific to their associated research.

- The web server and database server are different machines. The database server is not accessible from the internet. The web service access to the database is using the least-privilege method of access. It only has read and write privileges to the database, no delete or alter. All free-form text fields are "cleaned" before being sent to the SQL engine for access to the database. This makes SQL Injection much more difficult. Also, because of the least-privilege access it would be nearly impossible for a hacker to do any damage to the data since the web server does not have any administrative privileges to the database. Monthly archives are made off all servers and saved for an extended period of time. Backups are spot-verified. Server operating system software is patched as updates are made available. Server logs are monitored for system problems and hacking attempts. All critical STRONG STAR data transactions are maintained in a series of audit and archive tables. Every member of the research team will be trained and monitored about how to handle and protect both medical and research records.

*Who will be responsible for ensuring research records are secured prior to destruction date?* Research staff named in the protocol and the University of Texas Health Science Center at San Antonio (UTHSCSA) STRONG STAR Data Core supporting this project.

*Destruction plan for research records: When and how will the research documents, data files, and the master code be destroyed?*

There is no plan to destroy research documents or data files. Participant identifiers and PHI for both mental health providers and patients seeking treatment will be destroyed upon closure of the study with the IRBs. Informed consent documents will be stored securely for three years following completion of the research; HIPAA authorizations will be stored for six years IAW Federal regulations. Video-recordings of training cases will be copied onto DVD at the individual performance sites, labeled with the providers study number, and mailed directly to the University of Pennsylvania Center for the Treatment of Anxiety for review as part of case consultation teleconferences using an express service (e.g., FedEx) and tracked for receipt. Following review with the participant provider, the tapes will be destroyed by the Center for the Treatment of Anxiety staff.

**Sensitive Research Information***

*Does this research involve collection of sensitive information about the subjects that should be excluded from the electronic medical record?*

No

*9.2. Subject Privacy*

Participants will be asked for consent for all study procedures. No procedures will be performed without prior consent of the participant. The link between study ID number and a participants' protected health information will be password protected, with limited access to study personnel. Only staff directly working on the study will interact with participants and their patients and have access to data. Participants will interact with study staff in private offices and on the telephone.

*9.3. Data Disclosure*

Data will not be disclosed to anyone who is not listed under Personnel. If information from this study is published or presented at scientific meetings, names and other personal information will not be used.

**Data Protection***

x Name Street address, city, county, precinct, zip code, and equivalent geocodes

x All elements of dates (except year) for dates directly related to an individual and all ages over 89

x Telephone and fax number

x Electronic mail addresses

Social security numbers

Medical record numbers

Health plan ID numbers

Account numbers

Certificate/license numbers

Vehicle identifiers and serial numbers, including license plate numbers

Device identifiers/serial numbers

Web addresses (URLs)

Internet IP addresses

Biometric identifiers, incl. finger and voice prints

Full face photographic images and any comparable images

Any other unique identifying number, characteristic, or code

None

**10. Consent**

*10.1. Consent Process*

10.1.1. Overview

Following recruitment, potential participants, both providers and treatment-seeking patients, who express an interest in participating in this research will have the study explained to them in a private and secure location by a member of the research team, the project coordinator, research assistant, or BOAs. The consent form and the HIPAA authorization will be reviewed in detail with potential participants and they will be given an opportunity to ask questions. However, it is also recognized that informed consent takes place as an ongoing dialogue between the investigator/study staff and participants during the entire duration of their participation.

**Children and Adolescents**

No children or adolescents will be enrolled in the study.

**Adult Subjects Not Competent to Give Consent**

All subjects must be competent to give informed consent.

*10.2. Waiver of Consent*

**Waiver or Alteration of Informed Consent***

No Waiver Requested

**Minimal Risk***

**Impact on Subject Rights and Welfare***

**Waiver Essential to Research***

**Additional Information to Subjects Written Statement of Research***

No

*If no written statement will be provided, please provide justification.*

**11. Risk / Benefit**

*11.1. Potential Study Risks*

With the handling of medical and research records, there is always the possibility of a breach of confidentiality. This risk may be experienced by both participating providers and patients.

Some patient-participants may develop mild to moderate temporary emotional discomfort associated with clinical interviews or filling out questionnaires.

*11.2. Potential Study Benefits*

Participating mental health providers will receive training in Prolonged Exposure (PE) by Dr. Foa and her team, the psychologists who developed the treatment and the world’s leader in the delivery of PE. They will also have the opportunity to receive continuing education credit without having to travel Patients seeking treatment for PTSD will be receiving treatment from a provider trained by the world’s leader in prolonged exposure and leading expert in the treatment of posttraumatic stress disorder.

*11.3. Alternatives to Participation (optional)*

Participation in the study is optional for both providers and patients. The decision to opt out or withdraw from the study will not affect a providers status in the military or as a civilian or any benefits that he/she typically receives. Patients may also choose not to participate or leave the study at any time. The alternative to participating in this study of treatment outcomes is not to participate; patients will receive clinical treatment regardless of whether they participate in this study or not. No matter what decision patients make, there will be no penalty to them and they will not lose any of their regular benefits. Leaving the study will not affect the medical or psychological care that patients receive.

*11.4. Data and Safety Monitoring*

Providers will conduct all safety monitoring typically done during behavioral therapies. The protocol coordinator, BOA, and research assistants will provide feedback to the providers of changes in symptoms noted on study measures that may need intervention by the provider. Data will be double-entered into the STRONG STAR database located at UTHSCSA for analysis.

*11.5. Risk / Benefit Assessment*

Study participants (providers): There are no anticipated risks for participating in the study. All participants will benefit from intensive training in an evidence-based treatment for PTSD. Study participants (patients): Patients are assessed in person by experienced BOAs who are skilled in dealing with any temporary distress the patient may experience during the assessments. The BOA will make efforts to help patients to feel as comfortable as possible (e.g., by giving breaks during the assessment, offering encouragement, encouraging participants to ask questions and to express discomfort if felt). Patients seeking treatment for PTSD may receive PE. PE is an efficacious and effective treatment for PTSD and associated problems. Therefore patients may experience a reduction in their symptoms. Rare, but Serious Risks: With the handling of research records there is always the possibility of a breach of confidentiality.
